# Li-Ion Conductivity of Single-Step Synthesized Glassy-Ceramic Li_10_GeP_2_S_12_ and Post-heated Highly Crystalline Li_10_GeP_2_S_12_

**DOI:** 10.1021/acsami.3c05878

**Published:** 2023-07-13

**Authors:** Xin Lu, Anna Windmüller, Dana Schmidt, Sandro Schöner, Chih-Long Tsai, Hans Kungl, Xunfan Liao, Yiwang Chen, Shicheng Yu, Hermann Tempel, Rüdiger-A. Eichel

**Affiliations:** † Institut für Energie- und Klimaforschung (IEK-9: Grundlagen der Elektrochemie), Forschungszentrum Jülich, Jülich, NRW 52428, Germany; ‡ Institut für Materialien und Prozesse für Elektrochemische Energiespeicher- und wandler, RWTH Aachen University, 52074 Aachen, Germany; § National Engineering Research Center for Carbohydrate Synthesis/Key Lab of Fluorine and Silicon for Energy Materials and Chemistry of Ministry of Education, Jiangxi Normal University, 330022 Nanchang, China

**Keywords:** ball milling, solid-state electrolyte, single step synthesis, superionic conductor, Li_10_GeP_2_S_12_

## Abstract

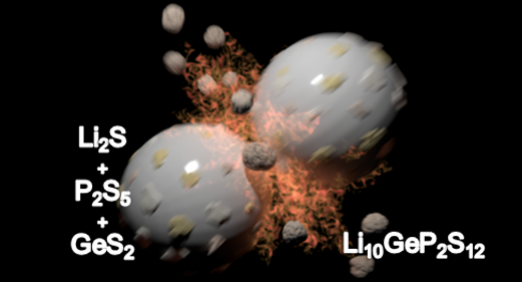

Li_10_GeP_2_S_12_ is a phosphosulfide solid electrolyte that exhibits exceptionally high Li-ion conductivity, reaching a conductivity above 10^–3^ S cm^–1^ at room temperature, rivaling that of liquid electrolytes. Herein, a method to produce glassy-ceramic Li_10_GeP_2_S_12_ via a single-step utilizing high-energy ball milling was developed and systematically studied. During the high energy milling process, the precursors experience three different stages, namely, the ‘Vitrification zone’ where the precursors undergo homogenization and amorphization, ‘Intermediary zone’ where Li_3_PS_4_ and Li_4_GeS_4_ are formed, and the ‘Product stage’ where the desired glassy-ceramic Li_10_GeP_2_S_12_ is formed after 520 min of milling. At room temperature, the as-milled sample achieved a high ionic conductivity of 1.07 × 10^–3^ S cm^–1^. It was determined via quantitative phase analyses (QPA) of transmission X-ray diffraction results that the as-milled Li_10_GeP_2_S_12_ possessed a high degree of amorphization (44.4 wt %). To further improve the crystallinity and ionic conductivity of the Li_10_GeP_2_S_12_, heat treatment of the as-milled sample was carried out. The optimal heat-treated Li_10_GeP_2_S_12_ is almost fully crystalline and possesses a room temperature ionic conductivity of 3.27 × 10^–3^ S cm^–1^, an over 200% increase compared to the glassy-ceramic Li_10_GeP_2_S_12_. These findings help provide previously lacking insights into the controllable preparation of Li_10_GeP_2_S_12_ material.

## Introduction

Since their first commercialization in 1991, Li-ion batteries have been used in more and more daily electronic devices and have become integral to modern society.^1^ However, the ever-increasing demand for higher energy storage and increased safety has accelerated the need for new battery technologies. All-solid-state batteries (ASSBs) have therefore been proposed as a replacement. To create a practical ASSB, a suitable solid-state electrolyte (SSE) with sufficiently high Li-ion conductivity is essential.^2^

The search for a suitable electrolyte to be used in ASSBs has led to a rich research history of solid-state electrolytes (SSEs). While sulfide-based SSEs have been in early development since the 1980s with the development of Li_4_SiS_4_,^3^ a large push in the development of lithium phosphosulfide electrolytes started around the new millennium.^4−6^ Due to the renewed interest in lithium phosphosulfide, a breakthrough was made with the synthesis of Li_10_GeP_2_S_12_, commonly referred to as LGPS.^7^ Li_10_GeP_2_S_12_ was synthesized by first mixing Li_2_S, P_2_S_5_, and GeS_2_ via vibration milling after which it was heat-treated at 550 °C in an evacuated quartz tube. Due to the high room temperature Li-ion conductivity achieved, in the range of 10^–2^ S cm^–1^, a large amount of research has subsequently been carried out on Li_10_GeP_2_S_12_ by various groups. Although various synthesis routes for Li_10_GeP_2_S_12_ have been proposed, they all typically follow a two-step regime in which the material is first milled after which a heat treatment process is carried out.^8−15^

This work begins by first presenting an alternative synthesis method, which is capable of producing glassy-ceramic Li_10_GeP_2_S_12_ via single-step high energy ball milling. While various lithium phosphosulfide-based solid electrolytes have been synthesized via continuous ball milling, none have so far been reported for the lithium superionic conductor Li_10_GeP_2_S_12_.^16−20^ In this work, stoichiometric ratios of Li_2_S, GeS_2_, and P_2_S_5_ were subjected to intensive kinetic collisions via high-energy ball milling. Investigations were then carried out after various continuous milling times utilizing powder X-ray diffraction (XRD). It was determined that three distinct stages could be identified and that with sufficient milling times, a glassy-ceramic Li_10_GeP_2_S_12_ could be achieved without a heat treatment step. Electrochemical impedance spectroscopy (EIS) was carried out to show that, despite its low crystallinity and high amorphous content, a high Li-ion conductivity was achieved with a value of 1.07 × 10^–3^ S cm^–1^ at room temperature.

In the second part of this work, the crystallinity of the as-milled Li_10_GeP_2_S_12_ was investigated utilizing transmission XRD with hard X-rays (HXRD) along with quantitative phase analysis (QPA) where it was shown to contain a large percentage of X-ray amorphous phases (44.4 wt % of amorphous content). The crystallinity of the as-milled Li_10_GeP_2_S_12_ was then increased by utilizing an additional heat treatment step. The heat treatment was carried out at various temperatures, and its X-ray amorphous and crystalline content was determined via QPA. Under optimal heat treatment conditions, the crystallinity of the heat-treated as-milled glassy-ceramic Li_10_GeP_2_S_12_ increased drastically, which results in an almost fully crystalline Li_10_GeP_2_S_12_. With the increase in crystallinity, it was found that the Li-ion conductivity also increased significantly by over 200% to 3.27 × 10^–3^ S cm^–1^ at room temperature for the optimal sample. However, it was also observed that a small change in the heat treatment temperature could drastically affect the amount of amorphous and crystalline phases, which in turn greatly affects the Li-ion conductivity. The work demonstrates a new single-step synthesis method capable of producing glassy-ceramics Li_10_GeP_2_S_12_ with high Li-ion conductivity as well as highlights the importance of temperature control in the post-heat treatment step to be able to achieve the highest crystallinity and even greater Li-ion conductivity.

## Results and Discussions

### Phase Evolution and Ionic Conduction of Li-Ge-P-S Prepared via High-Energy Ball Milling

To explore the high-energy ball milling synthesis of Li_10_GeP_2_S_12_, the rotational speed was fixed at 1500 rpm. The materials were loaded into the jar in stoichiometric ratios, as described in the experimental section. High-energy ball milling was carried out for up to 520 min, with structural and impedance characterization taken after various ball milling time intervals. The crystalline phases of the as-milled samples were determined via XRD using Cu-Kα radiation in reflection geometry. As sulfides are known to be highly sensitive to atmospheric conditions, Scotch Magic tape had to be used to protect the material from direct exposure to the atmosphere during XRD measurements. However, using this protective barrier is only effective within the first 2 h after exposure. Further, it induces a background and reduces the signal-to-noise ratio, making the determination of crystalline phases difficult. As such, the obtained diffractograms were treated by first determining and subtracting the background and K_α2_ intensities via HighScore Plus (Malvern Panalytical), after which the data were smoothed via the Savitzky–Golay method. While this method allows for the better qualitative determination of Bragg peaks, it removes any amorphous features but retains the information on crystalline phases.^21^ With this information, the phase evolution of Li_10_GeP_2_S_12_ during the high-energy ball mill is identified qualitatively.

**Figure 1:**
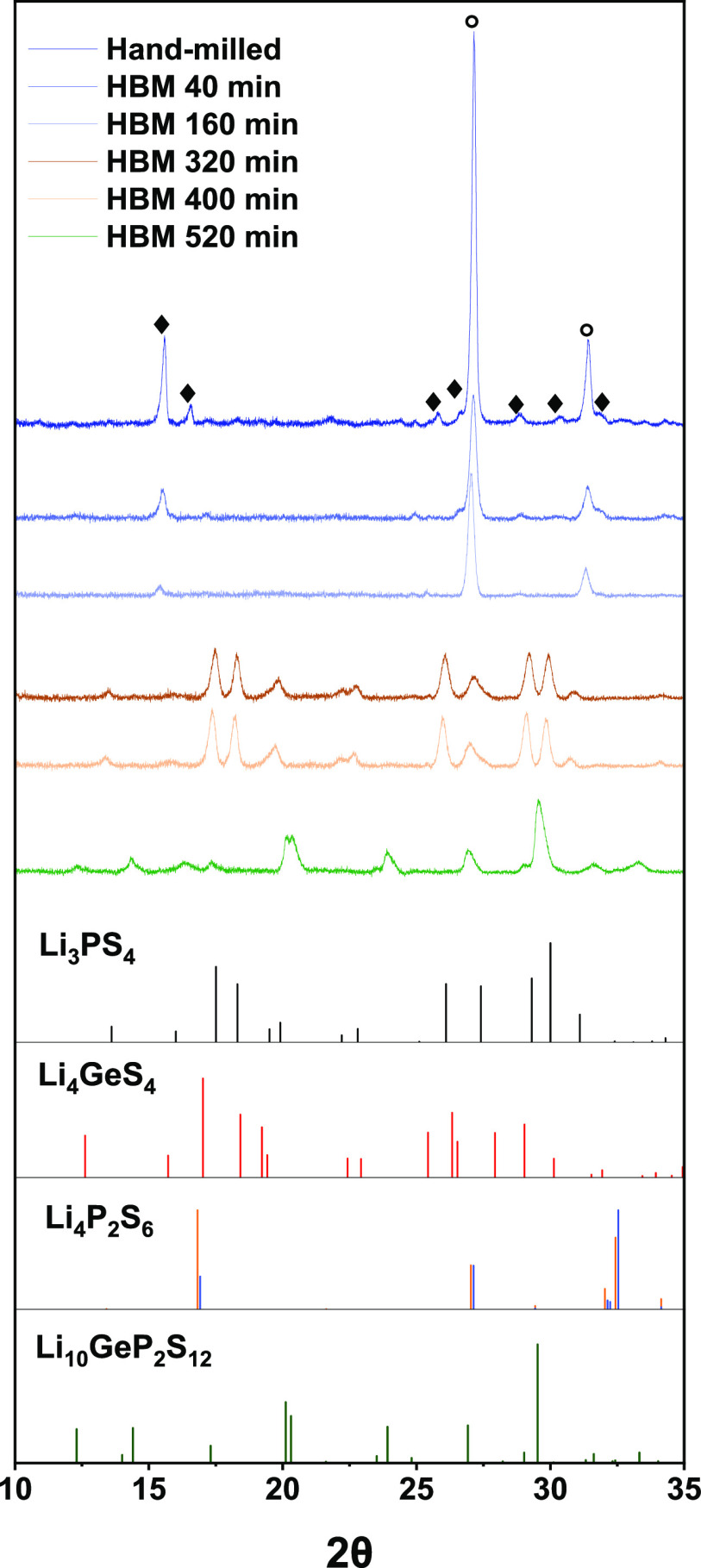
XRD patterns of Li-Ge-P-S samples prepared via HBM. Reference patterns are shown below. Circle indicates the peaks assigned to Li_2_S and diamond for GeS_2_. The references used to assign the reflections are Li_2_S (ICSD 54396),^22^ GeS_2_ (ICSD 44),^23^ Li_3_PS_4_ (ICSD 35018),^24^ Li_4_GeS_4_ (ICSD 95649),^25^ Li_4_P_2_S_6_ (ICSD 33506 (*P*6_3_*mcm*, in blue) and 242170 (*P–*31*m*, in orange)),^26,27^ and Li_10_GeP_2_S_12_ (ICSD 188887).^11^

**Figure 2:**
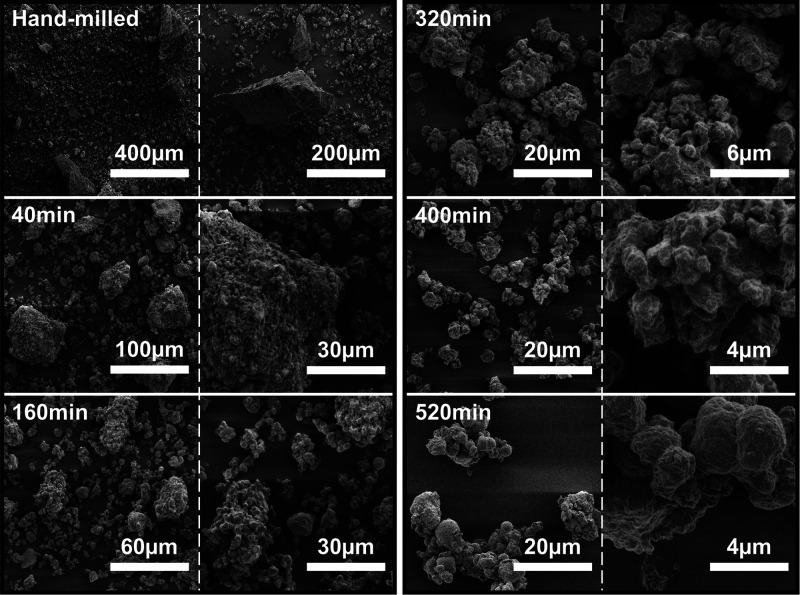
SEM images of Li-Ge-P-S samples of the hand-milled material before HBM and after HBM for 40, 160, 320, 400, and 520 min.

Figure 1 reveals changes in the crystalline phases of the raw precursors and powders after five different continuous milling durations. From the evolution of the crystalline phases, three distinct stages can therefore be determined. They are the ‘Vitrification zone’ from 0 to 160 min of milling, ‘Intermediary zone’ from 320 to 400 min of milling, and ‘Product Stage’ (520 min). The morphology of the material was determined as per the experimental section, with a particular focus placed on reducing alterations caused by the electron beam or environmental factors to produce a truer-to-life image (Figure 2).

The XRD patterns of the hand-milled sample show high-intensity Bragg peaks belonging to Li_2_S (27.15° and 31.40°) along with Bragg peaks with lower intensities belonging to GeS_2_ (15.60°, 16.55°, 25.80°, 26.60°, 28.80°, 30.40°, and 31.80°). Since the P_2_S_5_ used was mainly amorphous, it cannot be seen in the diffractogram. After high-energy ball milling of only 40 min, the crystalline precursors have already been amorphized, as can be concluded from their fading Bragg peak intensities. While both Bragg peaks of Li_2_S are still present, their intensities have significantly reduced, indicating a drastic reduction in their crystallinity. Furthermore, the Bragg peak at 16.55° belonging to GeS_2_ is no longer visible. This trend continues as the milling time is increased to 160 min, with all characteristic Bragg peaks of GeS_2_ disappearing except for the initial highest intensity Bragg peak at 15.60°. All these observations point to the fact that between 0 and 160 min of milling, the crystallinity of all precursors was quickly destroyed under high energy collision. At the same time, cold welding and powder particles fracture occur, which results in the excellent mixing of powders at a macrolevel and amorphization. This initial vitrification process is purely mechanically driven since the temperature during the process is far below traditional diffusion temperature and no melting should be occurring at all (melting points: Li_2_S, 938 °C; P_2_S_5_, 288 °C; GeS_2_, 800 °C). However, it is noted from the experiment that the melt temperatures match the order of amorphization since the precursors with lower melt temperatures were amorphized first. During this stage, a large number of defects, such as point, line, and surface defects, were created by the plastic deformation of the particles. With the increase in lattice defects, the activation energy of interdiffusion would be vastly reduced. Regarding amorphization, it has been well discussed concerning the mechanical alloying of crystalline powders into amorphous metals with similar or higher melting points.^28−30^

Observations of the morphological change in Figure 2 during the Vitrification zone show two main trends, reduction and homogenization of particle sizes. Beginning at the hand-milled sample, particle sizes vary significantly from 10 μm particles to large irregular particles upward of about 200 μm, with particles still possessing sharp morphologies. The wide range of particle sizes demonstrates that while hand-milling may be mixing the different components, it is unable to homogenize particle sizes. After high-energy ball milling for just 40 min, the previously observed large irregular particles are no longer present. The large irregular particles are now broken into smaller particles ranging from 30 to 70 μm. As the grinding media smash into the particles, particulates are coated on the surface of the grinding balls. When two balls collide, a portion of the mechanical energy will accumulate in the particles in the form of excess lattice defects. As milling time is increased, diffusion distance is vastly reduced, leading to chemical homogeneity and amorphization.^31,32^ The beginning of this process can be observed upon closer observation of the surfaces of the large particles. The small particulates of less than 5 μm are stacked on the surface of the large particles. As continuous cold-welding and fracturing occur, the smaller particles may be embedded into the big ones and fractured again similar to making dough. After 160 min milling, the particle size was further reduced to about 30 μm or less since the fracture rate is more extensive than cold-welding. Detailed observation on the particle surface shows that the particulates upon the surface of the particles no longer possess a well-defined crystalline appearance. Instead, the particulates now possess a smooth surface.

Between 320 and 400 min of milling (Intermediary zone), it is interesting to note that the original precursors disappear entirely. Instead, new crystalline phases are formed, predominantly Li_3_PS_4_ and Li_4_GeS_4_ (Figure 1). The XRD patterns at both 320 and 400 min show little difference. In both cases, the well-defined Bragg peaks are attributed to Li_3_PS_4_ (reference in black) and Li_4_GeS_4_ (reference in red). Morphological changes are shown in Figure 2. After 320 min milling, the maximum particle size is 15 μm, about half of the powder size at 160 min of milling. The surface of the particles milled for 320 min appears to be smooth, with small particulates fused with the surface. Increasing the milling time to 400 min led to a further reduction in particle size, with a maximum particle size now only about 5 μm. Similar to 320 min of milling, the smooth particles are coated with a sparse layer of particulates. With further increase in defect level due to continuous cold-welding and fracturing, the mixing is already at the atomic level. With heat assistance due to the accumulation of heat caused by each high-energy collision and friction between balls and powders, diffusion slowly occurred. As a consequence, new compounds were therefore formed. Because the formation of both Li_3_PS_4_ and Li_4_GeS_4_ are exothermic, their appearance would also ignite a faster reaction.

A few differences are noticed compared to intermediary phases of Li-Ge-P-S obtained previously synthesized via a heat treatment route.^21^ The samples synthesized via heat treatment have a few characteristic features, those being the presence of pin-holes and the presence of needle-like structures. These features are absent in the Intermediary zone of the samples obtained via high-energy ball milling. The lack of pin-holes could be attributed to the formation process of crystalline phases as formed via high-energy ball milling. The pin-holes are present due to the pressure build-up within the particle during heat treatment.^21^ However, during high-energy ball milling, the surface of the amorphous particles is subject to both intense forces and temperatures as the balls collide with each other. This collision will cause crystalline particulates to be formed at the surface of the particle instead of being precipitated from the interior of the particle; thus, no pressure buildup is observed, and pin-holes will not be generated. The high forces caused by the collision of the balls also prevent the formation of any needle-like structures. As collisions occur constantly, the crystalline particulates are likely embedded into the amorphous matrix moments after formation leading to an amorphous layer covering the surface due to the high forces experienced. This cycle of crystalline particulates being formed upon the surface before being quickly forced into the amorphous bulk and the surface returning to an amorphous nature is repeated continuously.

The final desired product Li_10_GeP_2_S_12_ is achieved after continuous high-energy ball milling for 520 min, as shown in Figure 1. The dominant Bragg peaks match that of the reference Li_10_GeP_2_S_12_ pattern along with Bragg peaks belonging to secondary phases Li_3_PS_4_ (17.35°, 29.00°, shoulder at 29.80°) and Li_4_P_2_S_6_ (16.40° and 32.50°). Since a typical successful formation of Li_10_GeP_2_S_12_ structure requires high temperatures upward of 600 °C, the formation of Li_10_GeP_2_S_12_ in the present study can be attributed to the combination of defect generation and the temperature due to collision. The morphology of the material formed (Figure 2) shows the appearance of smooth and rounded particles. The particulates upon the particles also no longer appear to be distinct instances. Instead, they are integrated with the particle.

**Figure 3:**
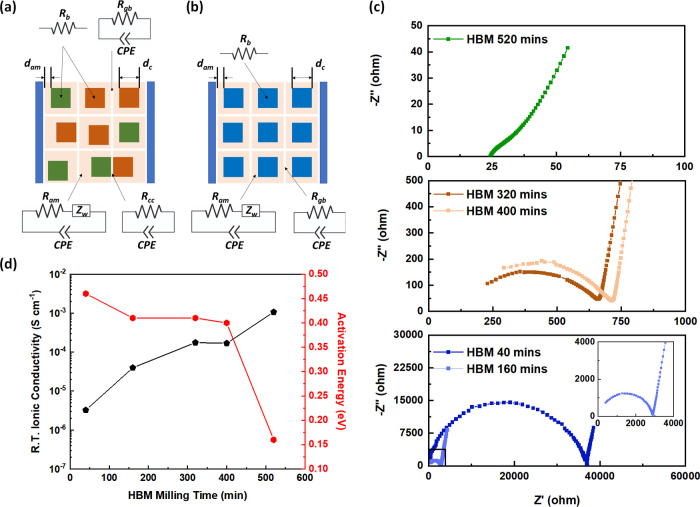
Schematic layout of solid electrolytes possessing a majority of (a) two crystalline phases and (b) a single crystalline phase. (c) Nyquist plots at room temperature of the materials after high-energy ball milling (HBM) at different times. Equivalent circuits and fitting results for all temperatures can be found in Figures S3, S6, S10, and Table S2-S6, respectively. (d) Room temperature ionic conductivities of materials after HBM along with activation energies calculated from 0–60 °C.

The ionic conductivity is an essential parameter for SSEs, and therefore it is evaluated via impedance measurements from 0 to 60 °C. Due to the multiphasic nature of the materials, complex equivalent circuits have to be used to evaluate contributions of impedance due to different components. Figure 3a,b provides information on the illustration of crystals in a 2D format based on the observations of XRD results, in which the prepared samples possess a majority of two crystalline phases (a) or a single crystalline phase (b). The blue, green, and brown squares represent three different crystalline materials and the beige represents the amorphous layer. Since the pellet was made of particles through cold isostatic pressing, there only exists a mechanical bonding between the particles. Therefore, the white lines indicate particle boundaries. Their respective equivalent circuits are placed next to those components indicated by arrows. Rationally combining these element circuits, the equivalent circuits for each sample are presented in Figures S3, S6, S10, and S13.

Looking at the total resistance, the material within the Vitrification zone (0–160 min of milling) has a room temperature ionic conductivity of 3.24 × 10^–6^ S cm^–1^ after 40 min of milling and 4.00 × 10^–5^ S cm^–1^ after 160 min as shown in Tables S2 and S3 in the Supporting Information. Within the Vitrification zone, the crystalline components as identified by XRD, Li_2_S and P_2_S_5_, are extremely poor Li-ion conductors. As such, the main contributing factor to the materials ionic conductivity would thus have to come from the amorphous component of the material. This is supported by the fact that the room temperature ionic conductivity increases by over a factor as the milling time increases from 40 to 160 min, with the increase in milling time leading to an associated amorphous fraction increase.

For the samples milled for 320 and 400 min in the Intermediary zone, two major crystalline compounds, Li_3_PS_4_ and Li_4_GeS_4_, are present as identified by XRD. Since two crystalline compounds are present together with a large amount of amorphous, there are four mechanisms contributing to the impedance, which are resistances due to the two crystalline, the amorphous itself, the grain boundaries, and the phase boundaries between two different crystalline compounds as shown in Figure 3a. Due to the large difference between the reported ionic conductivities of the compounds (∼10^–4^ S cm^–1^ for Li_3_PS_4_^33−35^ and ∼10^–7^ to 10^–4^ S cm^–1^ for Li_4_GeS_4_^6,36,37^), two assumptions had to be made in designing the equivalent circuit. First, the surface of the grain is an amorphous phase due to the milling, leading to a low grain boundary resistance (*R*_gb_). Second, on average, all grain boundaries are the same. Thus, the grain boundary contributions from Li_4_GeS_4_, Li_3_PS_4_, and the amorphous phase can be merged into a single *R*_gb_. Other than *R*_gb_, there are other contributors to the total impedance, namely, the bulk resistance of all phases combined (*R*_b_), the amorphous resistance (*R*_am_), and the resistance when two differing crystalline particles are in contact with each other (*R*_cc_). As the results show in the Supporting Information (Tables S4 and S5), there is a little discrepancy in the room temperature ionic conductivity in the intermediary zone with only a tiny drop from 1.70 × 10^–4^ S cm^–1^ (HBM 320 min) to 1.69 × 10^–4^ S cm^–1^ (HBM 400 min). This difference could be due to slight differences in the crystalline composition between the two milling conditions.

In the final Production stage, only one primary crystalline compound is present, Li_10_GeP_2_S_12_. As there is only one major crystalline component, the *R*_cc_ is ignored (Figure 3b). Due to the use of the high-energy ball mill in forming Li_10_GeP_2_S_12_, the particles should therefore consist of nanocrystalline material within an amorphous matrix. Hence, it can be considered as having no grain boundaries and therefore *R*_gb_ is also ignored. The room temperature total ionic conductivity of the sample milled for 520 min was measured to be 1.07 × 10^–3^ S cm^–1^, as shown in Table S6. The Nyquist plots at room temperature of the samples after high-energy ball milling are presented in Figure 3c. Based on these, the room temperature ionic conductivities of the samples are calculated and shown in Figure 3d, together with the activation energy calculated based on the impedance results from 0 to 60 °C (Tables S2–S6). During the Vitrification stage, the activation energy decreases from 0.46 eV until a plateau at 0.41 eV is reached. There is a minimal change occurring within the Intermediary stage, with the activation energy maintaining at about 0.41 eV until a drastic drop to 0.17 eV after forming the final product of Li_10_GeP_2_S_12_. This agrees well with a previous study in which it was found that Li_2_S-GeS_2_-P_2_S_5_ glass possesses a much higher activation energy when compared to that of the Li_10_GeP_2_S_12_ crystal.^38^

### Post-heating to Improve the Crystallinity and Ionic Conductivity

Meanwhile, it has been shown in this work that glassy-ceramic Li_10_GeP_2_S_12_ with sufficient room temperature ionic conductivity can be formed simply by just utilizing the high-energy ball milling alone. However, due to the use of high-energy ball milling synthesis, the crystallinity is expected to be poor with the as-milled material containing a large amorphous phase. In an effort to increase the crystallinity, heat treatment for the LGPS_520min_ sample was then carried out at three different temperatures of 575, 600, and 625 °C. After the heat treatment, it was found that the powders fused and densified into a compact pellet (Figure S8), thus necessitating the need to hand mill the pellets into loose powders before additional experiments could be conducted.

**Figure 4:**
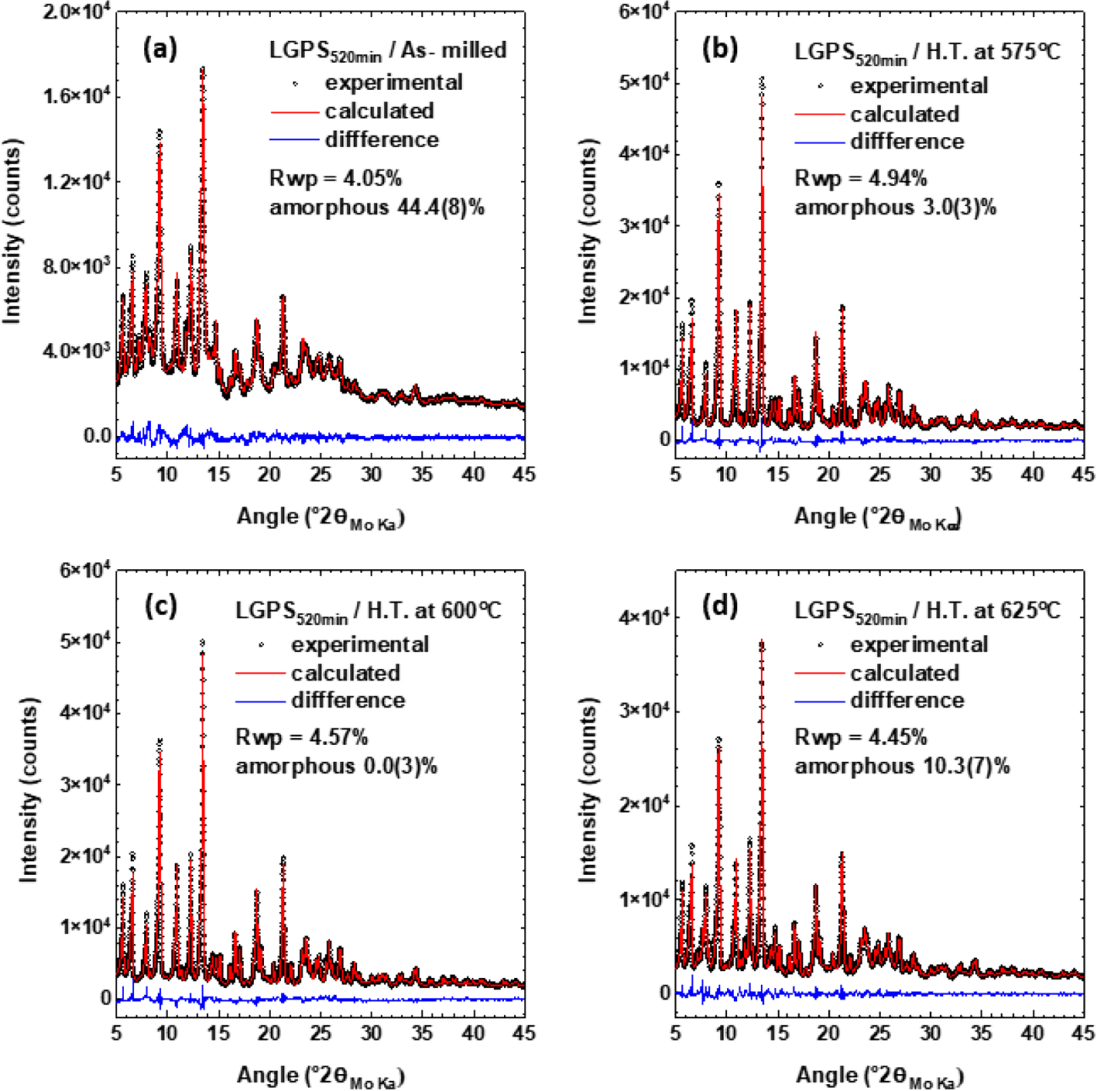
QPA results based on full-pattern fitting of HXRD data for (a) as-milled LGPS_520min_ as well as LGPS_520min_ samples after heat treatment at (b) 575 °C, (c) 600 °C, and (d) 625 °C. Reference patterns with relevant ICSD numbers are listed in Table 1.

Transmission HXRD along with QPA was carried out for the as-milled Li_10_GeP_2_S_12_ as well as the heat-treated samples loaded in sealed quartz capillaries. The QPA results are based on a full-pattern fitting method of HXRD data as described in detail in our previous study.^21,39^ In short, a combination of Rietveld and Pawley methods is used to describe the Bragg contributions and the contributions from amorphous contributions in the individual patterns to obtain a full pattern fit while the weight fractions of crystalline phases are corrected by a calibration factor (external standard method)^40^ to obtain absolute values for their weight fractions and derive the quantity of remaining non-crystalline/amorphous contributions. The results are provided in Figure 4 and Table 1.

**Table 1:** QPA Results Based on Full-Pattern Fitting of HXRD Data for As-Milled Li_10_GeP_2_S_12_ as Well as Samples after Heat Treatment at 575, 600, and 625 °C^a^

		mass fraction derived from QPA (wt %) of LGPS_520min_					
			heat treated				
material	structure	as-milled	575 °C	600 °C	625 °C	ICSD No.	ref
	amorphous	44.4(8)	3.0(3)	0.0(3)	10.3(7)		
GeS_2_	*P*1*c*1	0.46(7)				44	(23)
	*P*4_2_*/nmc*	0.29(2)				199200	(41)
	*P*12_1_*/c*1				1.47(6)	1947	(42)
	*I*4_1_*/acd*	0.00(4)				85527	(43)
Li_3_PS_4_	*Pnma*	9.0(7)		4.52(9)	14.6(5)	35018	(24)
Li_4_GeS_4_	*Pnma*	1.72(8)				95649	(25)
Li_4_P_2_S_6_	*P*6_3_*mcm*	3.29(10)	4.33(9)	4.35(9)	2.6(3)	33506	(26)
	*P–*31*m*	0.77(8)				242170	(27)
	*P*3_2_1				1.66(5)	434755	(44)
Li_10_GeP_2_S_12_	*P*4_2_*/nmc*	40.0(3)	92.6(2)	91.1(2)	68.9(3)	188887	(11)

a Reference patterns with relevant ICSD numbers are listed.

The HXRD pattern for the sample heat-treated at 600 °C can be described by three crystalline phases (Li_10_GeP_2_S_12_, Li_3_PS_4_, and Li_4_P_2_S_6_). Furthermore, the background in this dataset can be described by the same model background, as was used for fitting an empty capillary (Figure S9). The fit achieves a weighted pattern residual of 4.57% (Figure 4c) considering only the three crystalline phases and the model background. This suggests the absence of any X-ray amorphous contributions from the sample to the pattern. While the external standard method requires a highly crystalline standard to be measured under the same conditions as the samples, we took advantage of the high crystallinity of the sample that we achieved at 600 °C and used it as the “external standard” for quantifying the non-crystalline fractions in the other samples from the series.^40^

As shown in Table 1, it was revealed via QPA that the as-milled sample had a high X-ray amorphous content of 44.4 wt % along with impurities totaling 15.5 wt %. With the addition of heating to 575 °C for 8 h, the X-ray amorphous content drops significantly to only 3.0 wt % with germanium-based impurities disappearing and the desired phase, Li_10_GeP_2_S_12_ spiking massively to 92.6 wt %. With heating to 600 °C, the percentage of Li_10_GeP_2_S_12_ did not increase compared to LGPS_520min_ heat-treated at 575 °C. However, the phosphorus-based impurities rise from 4.33 to 8.87 wt %. It should be noted that most preparation methods of Li_10_GeP_2_S_12_ would create small amounts of impurities.^9,12,45,46^ The material starts to decompose at 625 °C with an increase of the X-ray amorphous phase to 10.3 wt %. Despite the slight increase in temperature of only 25 °C, the amount of impurities increased from 8.87 to 20.33 wt %.

**Figure 5:**
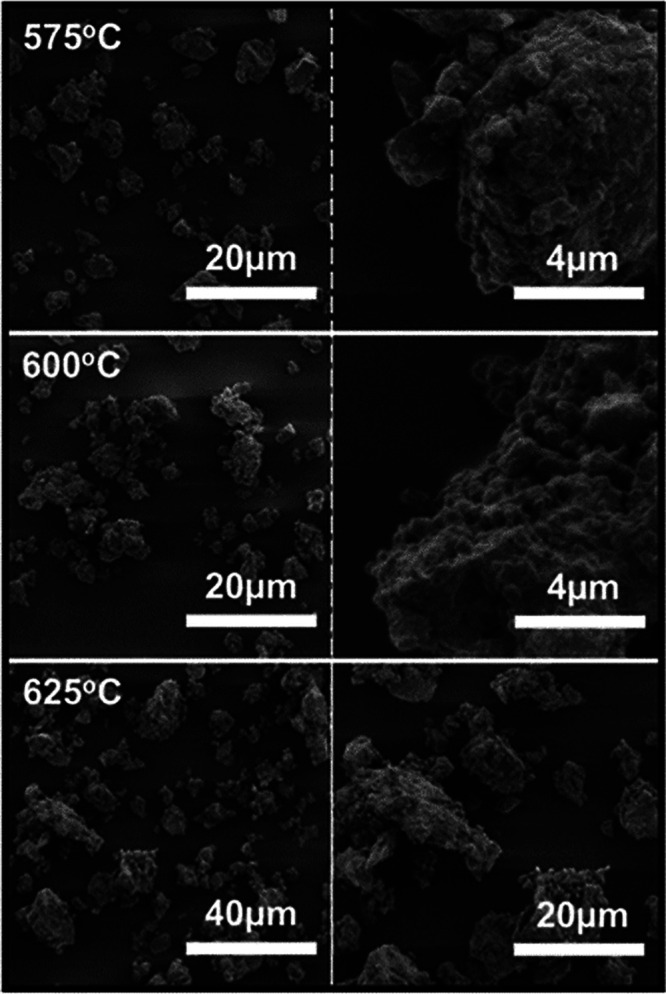
SEM images of the Li-Ge-P-S sample after 520 min of HBM with additional heat treatment at 575, 600, and 625 °C. Samples of heat-treated material were first broken apart via hand milling for 15 min, as after heat treatment, the powders densified and formed a solid puck, as shown in Figure S9.

SEM images of the heat-treated samples were recorded and presented in Figure 5. As previously mentioned, the samples had to be ground after heat treatment to receive a powder. After heat treatment at 575 °C, the previously smooth surface has particulate growth all over the surface. This appearance is further exaggerated at 600 °C, where the entire surface is covered by particulate growth. The growth at 600 °C has a crystalline appearance and a slightly enhanced size compared to that of 575 °C. These particulates are most likely the precipitated highly crystalline Li_10_GeP_2_S_12_. At 625 °C, the formation of large particulate covered flakes upward of 20 μm can be observed. These flakes are due to the decomposition of Li_10_GeP_2_S_12_ and formation of decompositional products.

**Figure 6:**
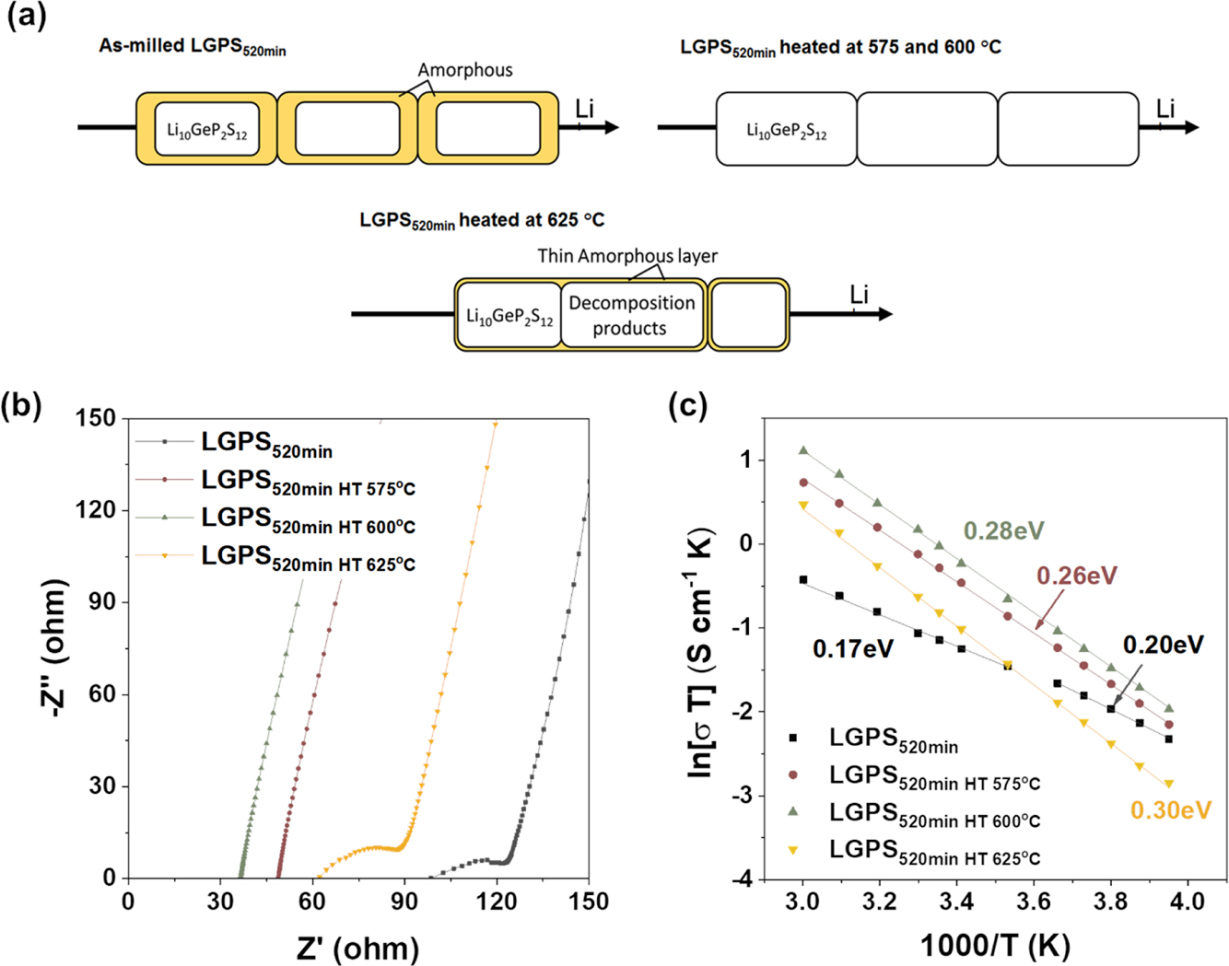
(a) Schematic diagrams of the Li-ion transport pathway before and after heat treatment. (b) Nyquist plots showing the room temperature impedance behavior of LGPS_520min_ without heat treatment and after heating at 575, 600, and 625 °C. Equivalent circuits for fitting the data can be found in Figures S6, S10, and S13. (c) Arrhenius plots of LGPS_520min_ before heat treatment and after heat treatment at 575, 600, and 625 °C measured over a temperature range of −20 to 60 °C.

The ionic conductivity of LGPS_520min_ before and after heat treatment was measured over a wide temperature range between −20 and 60 °C. As shown in Figure 6a, before heat treatment, the LGPS_520min_ consists of a large portion of the amorphous phase, and the sample is glassy-ceramic. Therefore, the Li-ions are likely required to conduct through both the crystalline Li_10_GeP_2_S_12_ particles and the amorphous boundary. After heating at 575 and 600 °C, the amorphous phase content drastically reduced. As the Li_10_GeP_2_S_12_ particles are bonded mechanically, the dominant conduction channel should occur through the Li_10_GeP_2_S_12_ particles. The sample heated at 625 °C contains about 10 wt % of the X-ray amorphous phase and 20 wt % of crystalline impurities. The Li-ion conduction pathway thus should need to pass through the thin amorphous layer, crystalline Li_10_GeP_2_S_12_, and side products. Based on these assumptions, the Nyquist plots of the samples are fitted with different equivalent circuits as detailed in the Supporting Information (Figures S6, S10, and S13). The experimental and fitting data match well, and the fitting results are summarized in Tables S6–S9 with the ionic conductivities calculated from the total resistances.

Nyquist plots of the samples measured at room temperature are given in Figure 6b. Accordingly, the ionic conductivity of the 575 °C heat-treated sample is more than double of the non-heat-treated sample, at 2.53 × 10^–3^ and 1.07 × 10^–3^ S cm^–1^, respectively. This is attributed to the fact that after heat treatment at 575 °C, the crystalline lithium superionic conductor, Li_10_GeP_2_S_12_, is doubled from 40 to 92.6 wt %, leading to a massive improvement in performance. After heat treatment at 600 °C, the room temperature ionic conductivity further increases by about 30% to 3.27 × 10^–3^ S cm^–1^. While the amounts of Li_10_GeP_2_S_12_ and Li_4_P_2_S_6_ are similar to that of 575 °C, the X-ray amorphous content has reduced from 3.0 wt % to an undetectable amount; in addition, an equal amount of Li_3_PS_4_ is present. While the difference from 3.0 wt % of X-ray amorphous content in the 575 °C sample to 0.0 wt % in the 600 °C can be considered extremely small, previous studies on a similar sample system have shown that small changes in the amorphous contents have a significant impact on the ionic conductivity of LGPS samples.^47^ Hence, the observed changes in ionic conductivity are in line with our observed changes in amorphous content. We conclude that the increase in ionic conductivity from 575 to 600 °C could be due to the reduction in amorphous content and an increase in the highly ionic conductive Li_3_PS_4_, indicating that even a small amount of amorphous content has a detrimental effect on the ionic conduction. As decomposition starts at 625 °C, the room temperature ionic conductivity drops to 1.48 × 10^–3^ S cm^–1^. Although the performance has degraded compared to the ideal 600 °C heat-treated sample, it is still ∼40% higher than the non-heat-treated LGPS_520min_ at room temperature. While it has been proposed from computational results that nano-crystallites surrounded by an amorphous structure, such as the as-milled LGPS_520min_, may perform better than highly crystalline Li_10_GeP_2_S_12_, we have shown that this may not be the case in practicality.^48^ This disagreement is likely due to the method in which our nano-crystallites are formed, leading to many undesired side products reducing our performance. However, our results agree with a recent experimental study that synthesized crystalline Li_10_GeP_2_S_12_ via a conventional solid-sate route and then ball-milled the crystalline material to produce nano-crystallites next to amorphous regions. The authors found that the nanosizing and disordering, when realized through ball milling, also reduce the migration dynamics of Li-ions.^47^

The Arrhenius plots and the calculated activation energies of the samples are shown in Figure 6c. It can be seen that the heat-treated samples seem only to have a single activation energy over −20 to 60 °C. In comparison, the as-milled sample has an activation energy at the low-temperature regime from −20 to 0 °C and another activation energy at the high-temperature regime from 20 to 60 °C. The activation energies of the samples increase as the heat treatment temperature increases. Starting from 0.26 eV for the sample heated at 575 °C, the activation energy expands to 0.28 eV for the 600 °C treated sample and consequently to 0.30 eV for the sample heated at 625 °C. The as-milled LGPS_520min_ possesses an activation energy of 0.20 eV at low temperatures, which reduces to 0.16 eV at a high-temperature regime.

Although multiple groups have characterized the lithium transport pathways in glassy-ceramic Li_10_GeP_2_S_12_, the exact mechanism is still up for debate.^9−11,49−51^ Several theories have been proposed in the literature to explain the change of activation energy in the high-temperature and low-temperature regimes. This includes the precipitation of blocking grain borders, which may influence ion transport at lower temperatures as well as the presence of a diffuse phase transition. Additional possibilities may involve modifying the rate-limiting transport step along with transitioning from a quasi-one-dimensional to three-dimensional transport at high temperatures. Meanwhile herein, the as-milled LGPS_520min_ contains multiphase side products (∼15 wt %) and a similar amount of crystalline Li_10_GeP_2_S_12_ (40 wt %) and X-ray amorphous phases (44.4 wt %), which could be the reason for the different activation energies in different temperature ranges as the Li-ion transportation pathways are complex and could significantly be influenced by temperature.

## Conclusions

This work demonstrates that the glassy-ceramic Li_10_GeP_2_S_12_ can be achieved by a single-step synthesis via high-energy ball milling for 520 min. Three stages of the formation of glassy-ceramic Li_10_GeP_2_S_12_ have been identified, the ‘Vitrification zone’, during which time the material is homogenized and increases its amorphous content, the ‘Intermediary zone’, during which two compounds, Li_4_GeS_4_ and Li_3_PS_4_, are formed, and the ‘Product stage’ during which the desired glassy-ceramic Li_10_GeP_2_S_12_ is formed. Equivalent circuits were designed taking into account information provided by XRD to evaluate the impedance results of the samples obtained at different stages. By utilizing transmission HXRD combined with quantitative phase analyses, it was found that the glass–ceramic Li_10_GeP_2_S_12_ achieved via single-step synthesis despite its low crystallinity possessed sufficient room temperature ionic conductivity 1.07 × 10^–3^ S cm^–1^. To improve the crystallinity and ionic conductivity, post-heating was carried out on the glassy-ceramic Li_10_GeP_2_S_12_ at various temperatures. The best-performing Li_10_GeP_2_S_12_ was achieved after heat treatment of 600 °C possessing significantly improved crystallinity and a room temperature ionic conductivity of 3.27 × 10^–3^ S cm^–1^, about a 200% increase over the as-milled. It was also found that even a slight increase in heating temperature will lead to pronounced decomposition of LGPS. Overall, a new single-step method of producing the glass-ceramic Li_10_GeP_2_S_12_ has been realized. The crystallinity and ionic conductivity of the glass-ceramic Li_10_GeP_2_S_12_ can be further improved significantly by heating for a short time at 600 °C.

## Experimental Methods

### Material Preparation

To prepare the material for high-energy ball milling, lithium sulfide (Li_2_S, 99.98%, Merck), phosphorus pentasulfide (P_2_S_5_, 99%, Merck), and germanium disulfide (GeS_2_, 99.99%, BOC Sciences) were first mixed in a pestle and mortar for 15 min at a molar ratio of 5Li_2_S:GeS_2_:P_2_S_5_ for a total weight of 5 g. This mixture was then placed into an airtight stainless steel milling jar of volume 50 mL with an inner lining made of Zirconium dioxide (Retsch). The jar was preloaded with 125 g of YTZ zirconia grinding media, which consists of eight pieces of Ø = 10 mm balls with the rest of the weight filled with Ø = 3 mm balls (Nikkato Corporation). All the processes were carried out in an argon-filled glovebox with O_2_ and H_2_O levels under 0.1 ppm.

Ball milling was carried out using a high-energy ball mill (Emax, Retsch) at 500 rpm for an hour, after which the speed was increased to 1500 rpm for a variable amount of time. Ball milling at 1500 rpm is carried out in 40 min segments with each segment consisting of 19 min of ball milling followed by 1 min of rest after which the milling direction is reversed and followed by another 19 min of ball milling and 1 min rest after which the next segment begins. After the completion of the milling process, the jars were brought back into the glovebox, where the materials were sieved from the grinding media. In the case of the investigation of the heating effects, 0.5 g of the as-milled powder was loaded into a 30 mL quartz tube and sealed using a plasma sealer (Plasmajet C2) within the glovebox. The sealed quartz tube then undergoes heat treatment in a muffle furnace (Nabertherm LT 9/13/b180) at various temperatures for 8 h with a ramping rate of 2 °C min^–1^ after which the quartz tube was cooled to room temperature passively.

### XRD Measurements

All sample preparations for all measurements were carried out in an argon filled glovebox. Reflection XRD on the powdered samples was carried out using an X-ray diffractometer (EMPYREAN, Panalytical) operating with Cu-Kα radiation in Bragg–Brentano geometry. The diffractograms were collected in a 2θ range from 10° to 35° at 45 kV and 40 mA with a step size of 0.004° and an effective counting time of 10.16 s per step with three scans for each sample. The samples were prepared by placing the powder onto a SiO_2_ substrate before being sealed with Scotch Magic tape to avoid air contamination.

Transmission HXRD measurements were performed to allow for quantitative phase analyses. The measurements were performed utilizing the same instrument as for reflection XRD with the configuration changed to a Mo Kα source in Debye–Scherrer geometry and a tension of 50 kV and 50 mA. The measurements were carried out from 5° to 60° with a step size of 0.008° and effective counting time per step of 80.645 s with 10 scans per sample, taking 12 h per measurement. To protect the sample against air contamination over such a long period, all the samples were heat-sealed in quartz capillaries (outer diameter = 1 mm, wall thickness = 0.01 mm). The Diffrac.Topas (Bruker) software package was used to perform the aforementioned quantitative phase analyses.

### SEM Measurements

Scanning electron microscope measurements were carried out to determine the morphological characteristics of the samples utilizing a Quanta FEG 650. To provide a true-to-life image, no conductive layer was applied. Due to the poor electric conductivity of the samples, the measurements used a combination of low acceleration voltage (1 kV), together with a fast scanning speed (1 μs, 64X line integration) to avoid charging effects, which has been described in previous work.^52^ In addition, samples were transferred between the glovebox and SEM using an airtight transfer module (Kammrath & Weiss), thus ensuring no exposure to atmospheric conditions occurred.

### Electrochemical Tests

For impedance measurements, powders were first hand-ground inside an argon filled glovebox using an agate mortar and pestle for 15 min until a smooth consistency was achieved, after which the pellets for EIS were made by first forming the shape using a 13 mm die. The formed pellets were then vacuum sealed in polythene bags and removed from the glovebox, after which they underwent cold isostatic pressing (CIP) (14.25 kN, ramp rate 142.5 kN/min, hold time 30 s), producing a high-density pellet. The pellets were then polished using sandpaper to remove the pressed skin, and no additional heat treatment was carried out. A thin layer of gold was sputtered onto the pellets utilizing a sputter coater in the glovebox (108auto, Cressington). Swagelok-type cells constructed from stainless steel with two stainless steel plungers acting as blocking current collectors were used for the EIS measurements. Polishing and sputtering of the pellet together with cell assembly were carried out in an argon-filled glovebox. Data was then collected with a potentiostat (VSP 300, BioLogic) utilizing an excitation potential of 50 mV and frequency range between 7 MHz and 1 Hz. The temperature of the cell was controlled and kept stable via the means of an environmental chamber (LabEvent T/20/40/EMC, Vötschtechnik). Detailed procedures are provided in the Supporting Information.

## Supporting Information Available

Pellet dimensions; Nyquist plots and Arrhenius plots along with used fitting equivalent circuits; fitting results of EIS measurements; photographs of the sample before and after heat treatment; QPA of HXRD data for an empty capillary (PDF)
