# Supplementary material for: Li-Ion Conductivity of Single-Step Synthesized Glassy-Ceramic Li10GeP2S12 and Post-heated Highly Crystalline Li10GeP2S12
Source: ACS Appl Mater Interfaces. 2023 Jul 13;15(29):34973–82. doi: 10.1021/acsami.3c05878 (PMC10375472; doi:10.1021/acsami.3c05878)
Supplement: Supplementary file 1 — am3c05878_si_001.pdf [file am3c05878_si_001.pdf]

Supporting Information

Li-Ion Conductivity of Single-Step Synthesized Glassy-Ceramic $Li_{10}GeP_2S_{12}$ and Post-heated Highly Crystalline $Li_{10}GeP_2S_{12}$

Xin Lu[a,b,]**, *Anna Windmüller*[a], *Dana Schmidt*[a,b], *Sandro Schöner*[a,b], *Chih-Long Tsai*[a], *Hans Kungl*[a], *Xunfan Liao*[c], *Yiwang Chen*[c], *Shicheng Yu*[a,]**, *Hermann Tempel*[a], *Rüdiger –A. Eichel*[a,b]

[a]Institut für Energie- und Klimaforschung (IEK-9: Grundlagen der Elektrochemie), Forschungszentrum Jülich, 52428 Jülich, Germany

[b]Institut für Materialien und Prozesse für Elektrochemische Energiespeicher- und wandler, RWTH Aachen University, 52074 Aachen, Germany

[c]National Engineering Research Center for Carbohydrate Synthesis/Key Lab of Fluorine and Silicon for Energy Materials and Chemistry of Ministry of Education, Jiangxi Normal University, 330022 Nanchang, China

AUTHOR INFORMATION

Corresponding Author

*Xin Lu E-mail: x.lu@fz-juelich.de

*Shicheng Yu E-mail: s.yu@fz-juelich.de

For impedance measurements, pellets were prepared according to the processes stated in the main text, with the diameters and thicknesses presented in **Table S1**. After sputtering, the solid electrolytes are placed into a stainless steel Swagelok cell and sealed. To ensure further protection against atmospheric degradation, all joints are additionally sealed using hot glue.

The sealed cells are placed into an environmental chamber (LabEvent T/20/40/EMC, Vötschtechnik). Depending on the material, two temperature ranges were tested. For the materials that underwent high-energy ball milling (HBM) for 40 min, 160 min, 320 min and 400 min, the temperatures at which impedance spectroscopy was carried out was 0 °C, 10 °C, 20°C, 25 °C, 30 °C, 40 °C and 60 °C. For the materials that underwent HBM for 520 min as well as the heat treated (HT) materials, a wider temperature range was used, that is to say, -20 °C, -15 °C, -10 °C, -5 °C, 0 °C, 10 °C, 20°C, 25 °C, 30 °C, 40 °C and 60 °C. For both temperature ranges, the materials were first bought down to the lowest measurement temperature (0 °C or -20 °C) and held for 4.75 h before potential electrochemical impedance spectroscopy (PEIS) was carried out. For each increase of 5 °C, the cell was stabilized at that temperature for at least 1.75 h, and for each increase of 10 °C, the cell was stabilized at that temperature for at least 2.75 h.

For PEIS, all materials were tested utilizing an excitation potential of 50 mV and a frequency range between 7 MHz and 1 Hz. 20 points were taken per decade with 1 measure per frequency.

Table S1. Diameter and thicknesses of polished solid electrolyte pellets produced from cold pressing material that has underwent high-energy ball milling (HBM) or HBM and heat treatment (HT).

Sample	Diameter (mm)	Thickness (mm)
HBM 40 min	11.46	1.21
HBM 160 min	11.35	1.17
HBM 320 min	11.31	1.15
HBM 400 min	11.25	1.21
HBM 520 min	11.30	1.21
HBM 520min HT 575 °C	11.29	1.22
HBM 520min HT 600 °C	11.25	1.17
HBM 520min HT 625 °C	11.27	1.10

Nyquist plot fitting Due to the complex nature of the material that underwent HBM for 40 min (**Fig. S1**) and 160 min (**Fig. S2**), a varied mixture of both extremely poor Li-ion conductors and ionically conductive amorphous phases, the contributions to the resistance cannot be easily modeled. As such only the total resistance, R_{total}, as taken from 0 Ω to the inflection point is given.

Due to the similarities in chemistry as demonstrated by XRD (**Fig. 1**), for the materials that underwent HBM for 320 min (**Fig. S4**) and 400 min (**Fig. S5**) similar equivalent circuits were used. Two assumptions were made during the design of the equivalent circuits, 1) Surface of the grain is amorphous, due to the HBM, leading to a low grain boundary resistance (R_{gb}) but high amorphous resistance (R_{am}) 2) On average, all grain-boundaries are same. R_{gb} is the combination of all 3 types of grain boundaries present, Li_4GeS_4, Li_3PS_4 and Amorphous. R_b is the combination of the bulk resistances of Li_4GeS_4, Li_3PS_4 and amorphous phases. R_{cc} represents the resistance present when Li_4GeS_4 and Li_3PS_4 are in contact with each other. CPE_{DP} describes the diffuse layer resistance. As measurement temperatures increase, however, the Li-ion transport becomes faster thus leading to lower measured resistances making separation of different contributions difficult thus necessitating the use of multiple, simplified equivalent circuits (**Fig S3**).

For the materials that underwent HBM for 520 min (**Fig. S7**), from observation of the XRD and QPA (**Fig. 1**, **Table 1**), it is shown that a large minority of the material, ~40 wt. % is amorphous with the majority of the remaining being $Li_{10}GeP_2S_{12}$. Due to the use of HBM we assume the material is nano-crystalline in an amorphous matrix thus R_{gb}, the grain boundary resistance, will be minimal and is ignored. Thus the material is simply modeled with R_b, the bulk resistance of the crystalline $Li_{10}GeP_2S_{12}$ and R_{am}, the resistance contribution of the amorphous phase. The equivalent circuits are presented in (**Fig. S6**).

For the material that underwent HBM for 520 min plus an additional heat treatment at 575 °C (**Fig. S11**) and 600 °C (**Fig. S12**), XRD and QPA (**Fig. 1**, **Table 1**) showed them to be extremely similar, both consisting of relatively pure crystalline $Li_{10}GeP_2S_{12}$ with negligible amorphous phase. As such the same model was used for both consisting of the bulk resistance R_b and the grain boundary resistance R_{gb}. The equivalent circuits are presented in (**Fig. S10**).

XRD and QPA (**Fig. 1**, **Table 1**) reveal that the material that underwent HBM for 520 min plus an additional heat treatment at 625 °C possesses a small amount of amorphous content (~10 wt. %), a large amount of side products (~ 20 wt. %) with the remaining being crystalline $Li_{10}GeP_2S_{12}$. As the amount of amorphous phase is relatively low, the resistance put up should be negligible. Due to the high crystallinity both the grain boundaries between similar materials (R_{gb}) as well as when differing crystalline particles are in contact with each other (R_{cc}) would be the main bottleneck to Li-ion transport and thus the measured resistances.

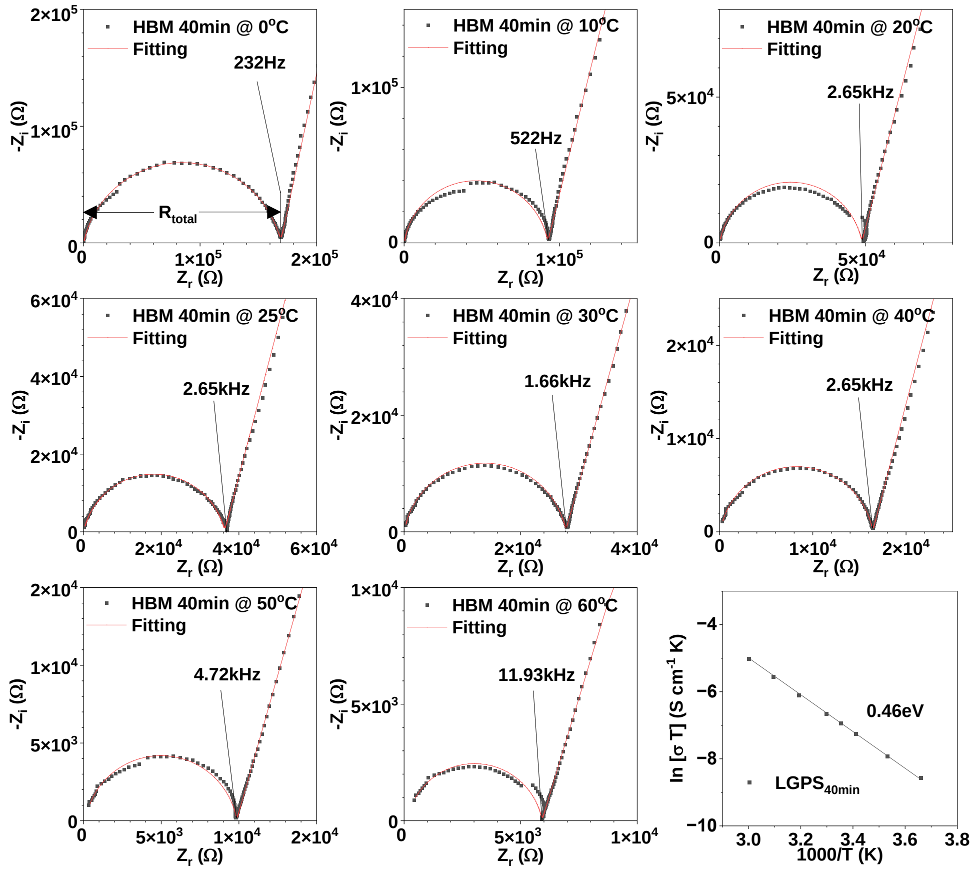

Figure S1. Nyquist plots for temperatures in the range between 0 °C and 60 °C and Arrhenius plot, with activation energy, of sample that has undergone HBM for 40 min.

Table S2. R_{total} and ionic conductivity measured from 0 °C and 60 °C for sample that has undergone HBM for 40 min (**Fig. S1**).

**(°C)**	**R_{total} (Ohm)**	**σ (S cm^{-1})**
**0**	169073	5.18e-7
**10**	92274	1.27e-06
**20**	48770	2.41e-06
**25**	36256	3.24e-06
**30**	27696.01	4.24e-06
**40**	16402.99	7.15e-06
**50**	9845.2	1.19e-05
**60**	5907.9	1.99E-05

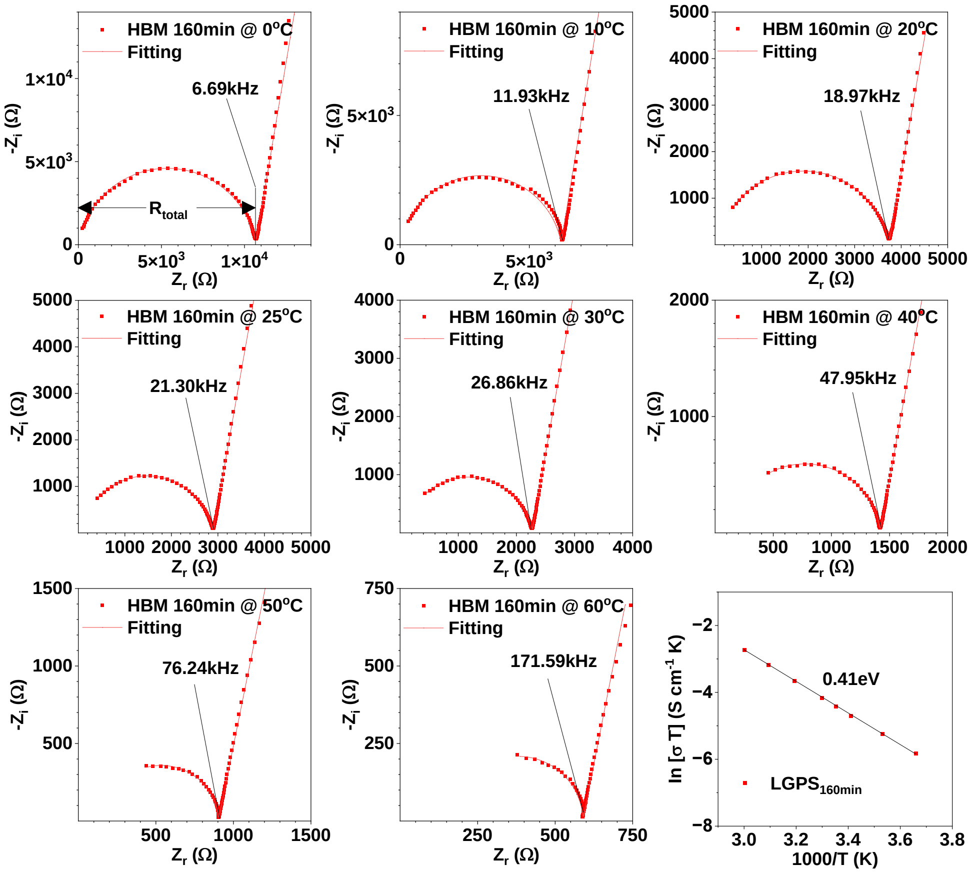

Figure S2. Nyquist plots for temperatures in the range between 0 °C and 60 °C and Arrhenius plot of sample, with activation energy, that has undergone HBM for 160 min.

Table S3. R_{total} and ionic conductivity measured from 0 °C and 60 °C for sample that has undergone HBM for 160 min (**Fig. S2**).

(°C)	R_{total} (Ohm)	σ (S cm^{-1})
**0**	10663.95	1.08e-05
**10**	6223.79	1.86e-05
**20**	3732.01	3.10e-05
**25**	2889.82	4.00e-05
**30**	2263.68	5.11e-05
**40**	1417.3	8.16e-05
**50**	902.3	1.28e-04
**60**	588.6	1.96e-04

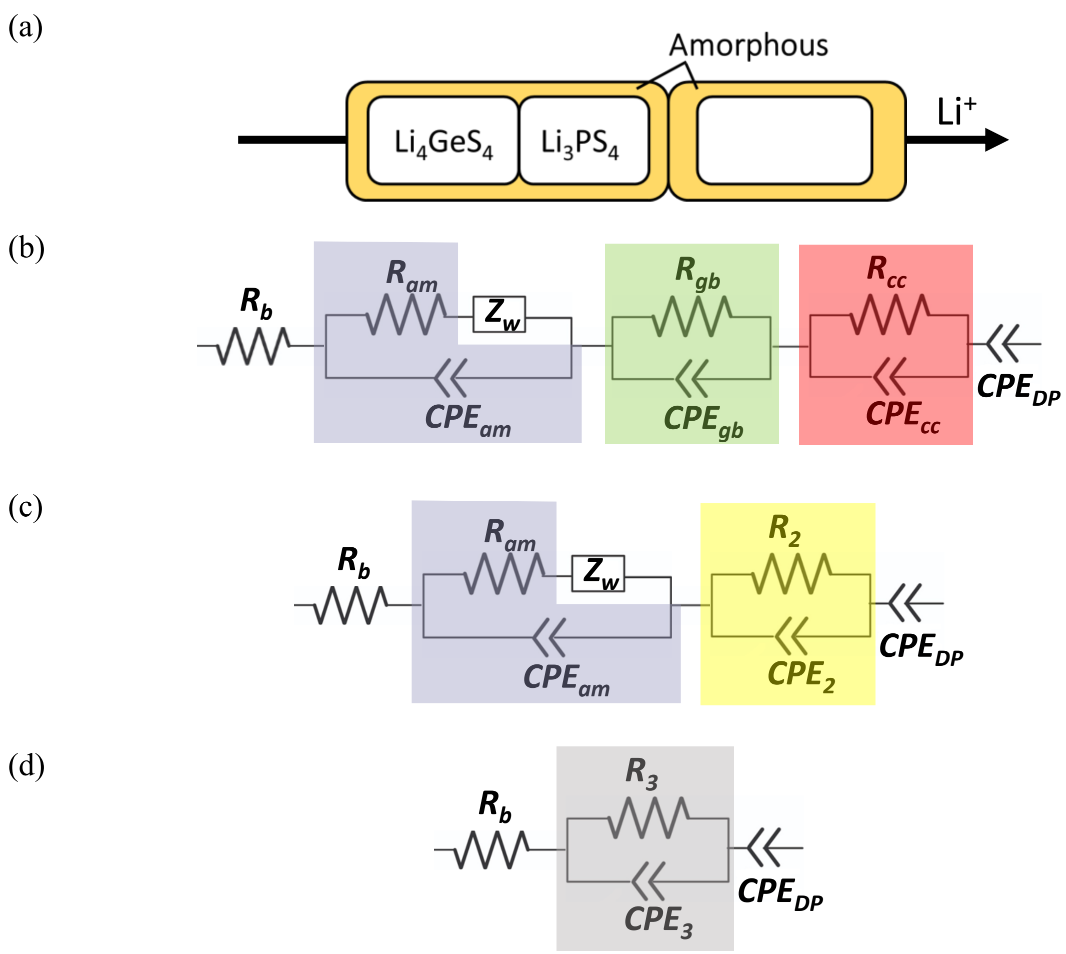

Figure S3. Model of Li-ion transport trough the material that has underwent HBM for 320 min (**Fig. S4**) and 400 min (**Fig. S5**) (a). Equivalent circuit used for fitting the impedance spectroscopy (b-d). For sample that underwent HBM for 320 min, (b) was used for 0 °C to 10 °C, (c) 20 °C to 30 °C and (d) 40 °C to 60 °C. For sample that underwent HBM for 400 min, (b) was used for 0 °C, (c) 10 °C to 20 °C and (d) 25 °C to 60 °C. Shaded portions of equivalent circuits are represented in the impendence spectroscopy (**Fig. S4** and **S5**) by semi circles of the same color. R_2 is the combination of both R_{gb} and R_{cc}, while R_3 is the combination of R_{am}, R_{gb} and R_{cc}. Both of these are used at higher temperatures where it becomes difficult to separate the contributions due to the increase in Li-ion transport speed leading to reduced measured resistances.

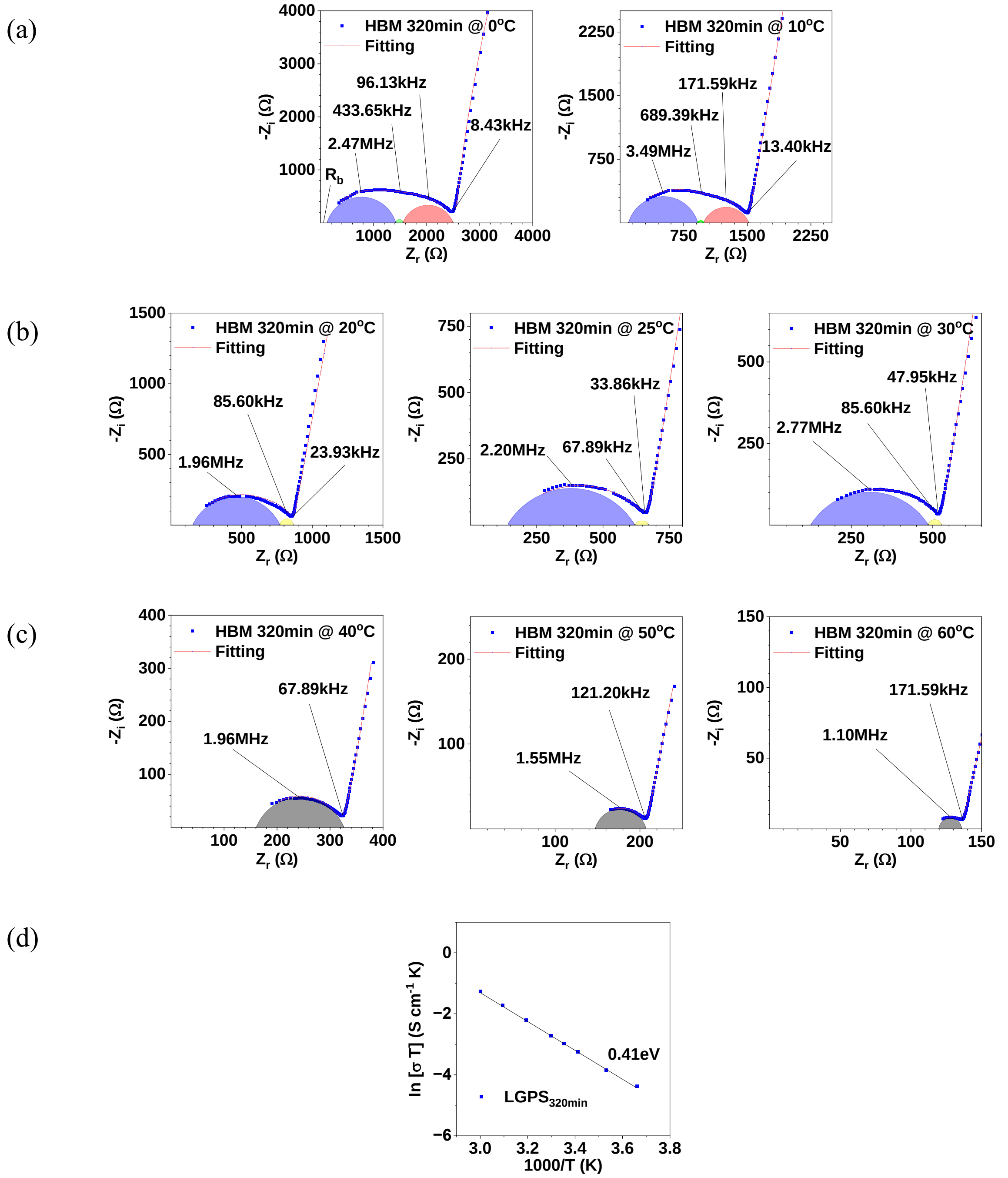

Figure S4. Nyquist plots of sample that has undergone HBM for 320 min from 0 °C to 10 °C (a), 20 °C to 30 °C (b) and 40 °C to 60 °C (c). Equivalent circuit used are found in **Fig. S3**. Arrhenius plot measured from 0 °C to 60 °C along with calculated activation energy (d).

Table S4. Fitting values used for sample that has undergone HBM for 320 min (**Fig. S4**). Where Q_{am} and a_{am} describe CPE_{am}, S_1 describes the Warburg element, Q_{gb} and a_{gb} describe CPE_{gb}, Q_{cc} and a_{cc} describe CPE_{cc}, Q_{DP} and a_{DP} describe CPE_{DP}, Q_2 and a_2 describe CPE_2 and Q_3 and a_3 describe CPE_3. R_{total} is the sum of all resistors in the circuit.

(°C)	R_b (Ohm)	Q_{am} (F.s^(a-1))	a_{am}	R_{am} (Ohm)	S_1 (Ohm.s^-1/2)	Q_{gb} (F.s^(a-1))	a_{gb}	R_{gb} (Ohm)	Q_{cc} (F.s^(a-1))	a_{cc}	R_{cc} (Ohm)	Q_{DP} (F.s^(a - 1))	a_{DP}	R_{total} (Ohm)	σ (S cm^{-1})
**0**	120.2	1.104e-9	0.822	1298	12.07	2.762e-8	1	133.7	1.926e-8	0.784	946.6	0.606e-6	0.898	2498.5	4.58e-05
**10**	99.18	1.311e-9	0.83	817.6	1	8.510e-9	0.773	67.39	3.573e-8	0.772	534.1	0.665e-6	0.898	1518.27	7.54e-05
	**R_b**	**Q_{am}**	**a_{am}**	**R_{am}**	**S_1**	**Q_2**	**a_2**	**R_2**	**Q_{DP}**	**a_{DP}**				**R_{total}**	**σ**
**20**	153.5	1.095e-8	0.733	617.6	1.568	1.254e-8	0.959	95.02	0.758e-6	0.887				866.12	1.32e-04
**25**	139.2	4.61e-8	0.666	481.2	1	6.219e-8	0.703	52.75	0.715e-6	0.905				673.15	1.70e-04
**30**	124.9	4.631e-8	0.651	361.7	1	3.533e-8	0.89	40.34	0.771e-6	0.902				523.94	2.18e-04
	**R_b**	**Q_3**	**a_3**	**R_3**	**Q_{DP}**	**a_{DP}**								**R_{total}**	**σ**
**40**	160.9	2.345e-8	0.766	165.2	0.928e-6	0.890								326.1	3.51e-04
**50**	147.3	2.026e-8	0.849	59.98	1.137e-6	0.880								207.28	5.52e-04
**60**	119.8	1.555e-8	0.969	16.11	1.514e-6	0.862								135.91	8.42e-04

(a)

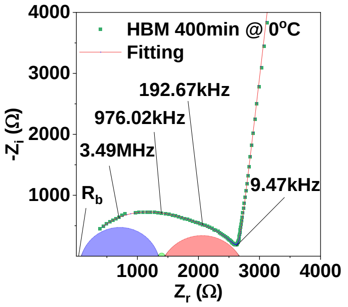

(b)

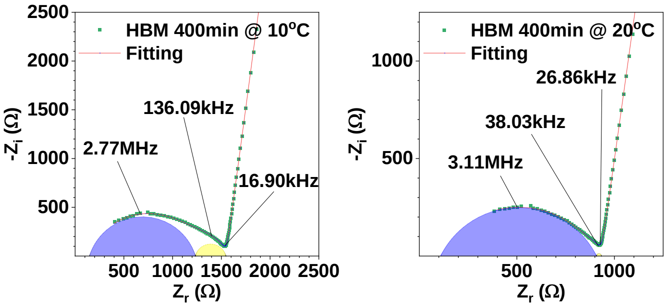

(c)

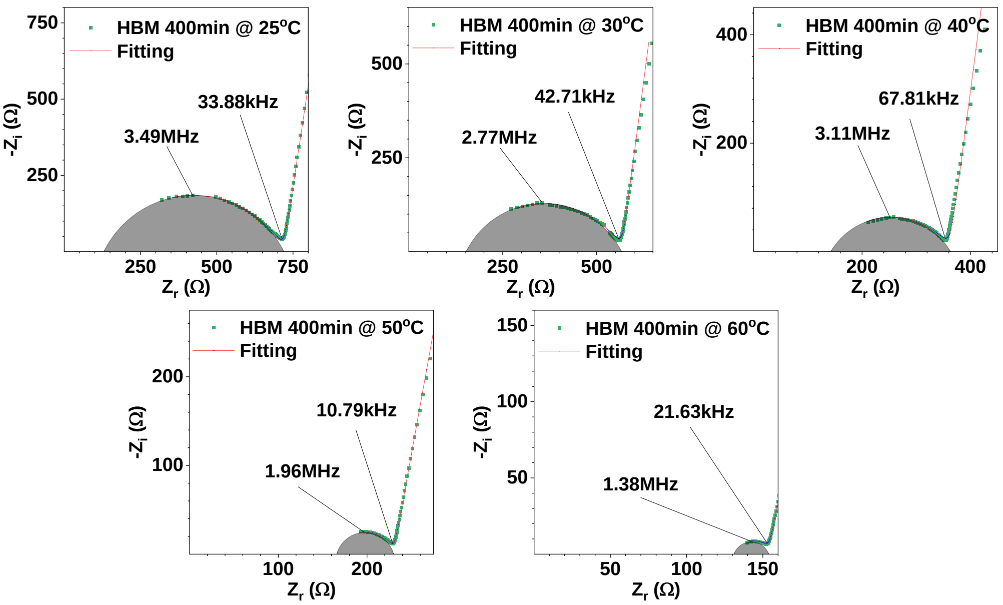

(d)

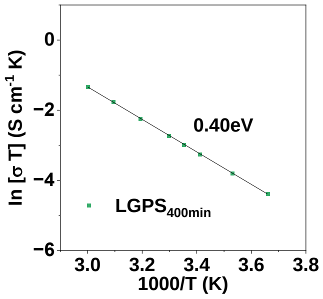

Figure S5. Nyquist plots of sample that has undergone HBM for 400 min at 0 °C (a), 10 °C to 20 °C (b) and 20 °C to 60 °C (c). Equivalent circuit used are found in **Fig. S3**. Arrhenius plot measured from 0 °C to 60 °C along with calculated activation energy (d).

Table S5. Fitting values used for sample that has undergone HBM for 400 min (**Fig. S5**). Where Q_{am} and a_{am} describe CPE_{am}, S_1 describes the Warburg element, Q_{gb} and a_{gb} describe CPE_{gb}, Q_{cc} and a_{cc} describe CPE_{cc}, Q_{DP} and a_{DP} describe CPE_{DP}, Q_2 and a_2 describe CPE_2 and Q_3 and a_3 describe CPE_3. R_{total} is the sum of all resistors in the circuit.

(°C)	R_b (Ohm)	Q_{am} (F.s^(a-1))	a_{am}	R_{am} (Ohm)	S_1 (Ohm.s^-1/2)	Q_{gb} (F.s^(a-1))	a_{gb}	R_{gb} (Ohm)	Q_{cc} (F.s^(a-1))	a_{cc}	R_{cc} (Ohm)	Q_{DP} (F.s^(a - 1))	a_{DP}	R_{total} (Ohm)	σ (S cm^{-1})
**0**	81.02	1.103e-9	0.819	1264	0.81	1.71e-8	1	104.8	6.206e-8	0.647	1219	4.633e-7	0.927	2668.82	4.56e-05
	**R_b**	**Q_{am}**	**a_{am}**	**R_{am}**	**S_1**	**Q_2**	**a_2**	**R_2**	**Q_{DP}**	**a_{DP}**				**R_{total}**	**σ**
**10**	147.4	1.3e-9	0.811	1087	1	2.22e-8	0.849	312.3	5.487e-7	0.914				1546.7	7.87e-05
**20**	109.2	8.452e-9	0.709	799.3	1	5.087e-8	1	24.79	5.99e-7	0.912				933.29	1.30e-04
	**R_b**	**Q_3**	**a_3**	**R_3**	**Q_{DP}**	**a_{DP}**								**R_{total}**	**σ**
**25**	130.5	1.005e-8	0.713	589.8	0.657e-6	0.906								720.3	1.69e-04
**30**	152.7	1.756e-8	0.710	413.5	0.699e-6	0.904								566.2	2.15e-04
**40**	142.4	7.18e-8	0.663	220.7	0.669e-6	0.919								363.1	3.35e-04
**50**	165.9	2.022e-8	0.834	64.07	0.988e-6	0.887								229.97	5.29e-04
**60**	131.1	0.141e-6	0.789	22.69	1.12e-6	0.887								153.79	7.91e-04

(a)

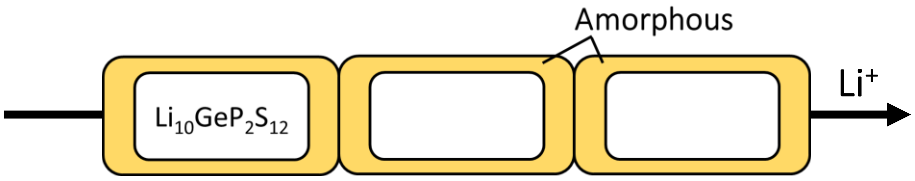

(b)

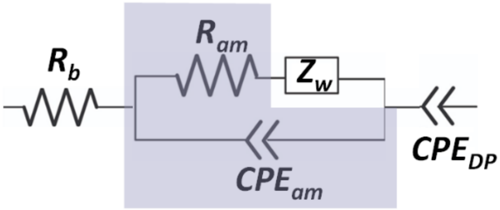

(c)

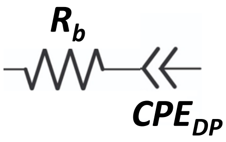

Figure S6. Model of Li-ion transport trough the material that has underwent HBM for 520 min (**Fig. S7**) (a). Equivalent circuit used for fitting the impedance spectroscopy (b-c). (b) was used for -20 °C to 30 °C, and (c) was used for -40 °C to 60 °C. Shaded portions of equivalent circuits are represented in the impendence spectroscopy (**Fig. S7**) by semi circles of the same color.

(a)

(b)

(c)

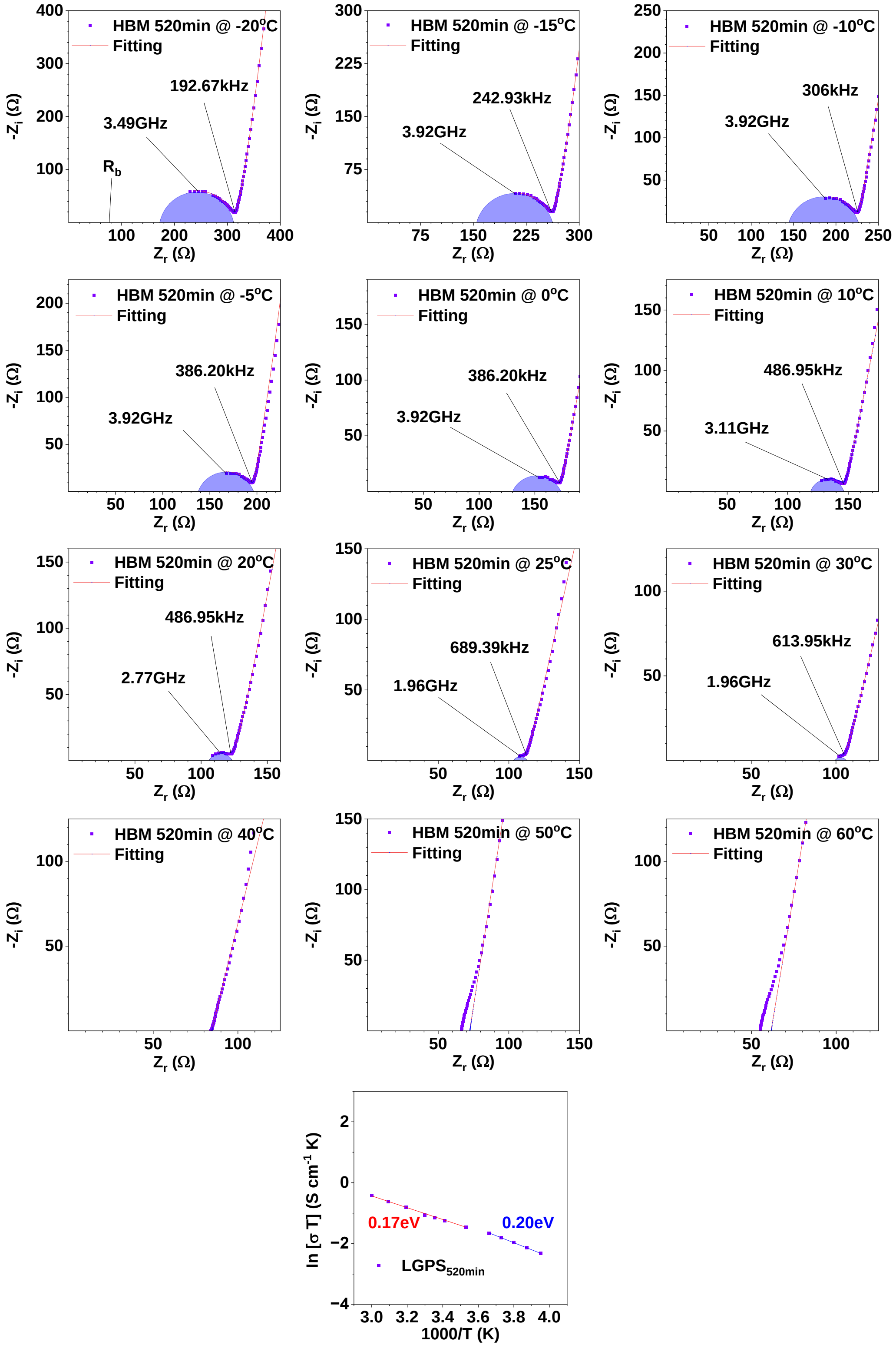

Figure S7. Nyquist plots of sample that has undergone HBM for 520 min from -20 °C to 30 °C (a), and 40 °C to 60 °C, the small hump present is attributed to the wire and ignored for the purposes of determining the resistance. (b). Equivalent circuit used are found in **Fig. S6**. Arrhenius plot measured from -20 °C to 60 °C along with calculated activation energy (c).

Table S6. Fitting values used for sample that has undergone HBM for 520 min (**Fig. S7**). Where Q_{am} and a_{am} describe CPE_{am}, S_1 describes the Warburg element and Q_{DP} and a_{DP} describe CPE_{DP}. R_{total} is the sum of all resistors in the circuit.

(°C)	R_b (Ohm)	Q_{am} (F.s^(a - 1))	a_{am}	R_{am} (Ohm)	S_1 (Ohm.s^-1/2)	Q_{DP} (F.s^(a - 1))	a_{DP}	R_{total} (Ohm)	σ (S cm^{-1})
**-20**	172.3	2.150e-9	0.878	139.4	4246	0.259e-6	0.950	311.7	3.87e-04
**-15**	154.7	5.079e-9	0.833	107.4	1112	0.384e-6	0.907	262.1	4.60e-04
**-10**	144.5	9.237e-9	0.812	81.91	381.1	0.407e-6	0.902	226.41	5.33e-04
**-5**	137.7	1.92e-8	0.782	58.7	71.63	0.386e-6	0.909	196.4	6.14e-04
**0**	130.0	5.073e-8	0.736	43.34	9.8e-11	0.487e-6	0.889	173.34	6.96e-04
**10**	119.0	2.596e-8	0.827	27.36	1005	0.519e-6	0.890	146.36	8.24e-04
**20**	106.0	0.201e-6	0.712	17.45	712.6	0.705e-6	0.864	123.45	9.77e-04
**25**	103.3	1.659e-6	0.652	9.65	1.086	0.731e-6	0.860	112.95	1.07e-03
**30**	99.5	3.146e-6	0.659	6.40	63.72	0.805e-6	0.853	105.9	1.14e-03
	**R_b**	**Q_{DP}**	**a_{DP}**					**R_{total}**	**σ**
**40**	84.48	5.490e-6	0.847					84.48	1.43e-03
**50**	72.26	0.547e-6	0.901					72.26	1.67e-03
**60**	61.56	0.799e-6	.895					61.56	1.96e-03

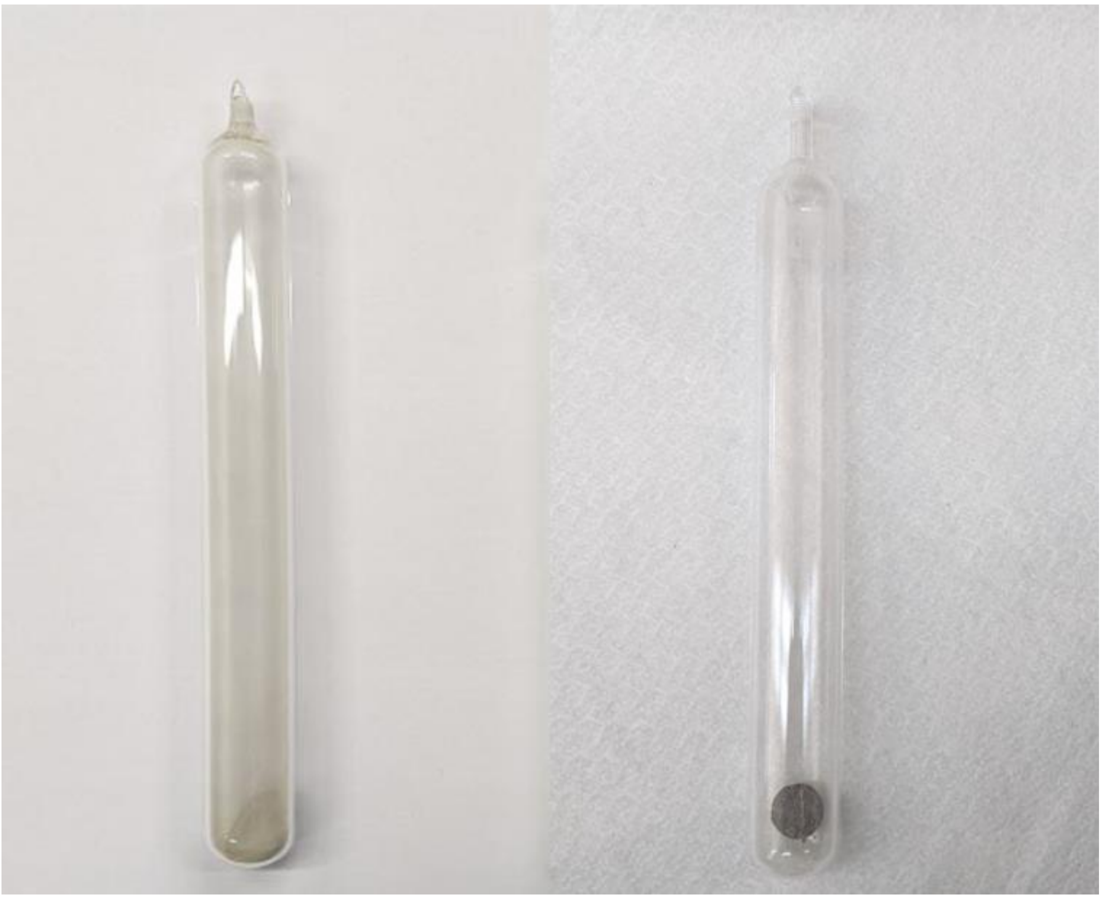

Figure S8. Sealed sample of $LGPS_{520min}$ showing its loose powdery form on the left. On the right is $LGPS_{520min}$ after heat treatment at 600 °C showing how the powders densified and forming a solid puck thus requiring hand milling before SEM or XRD is possible. All heat treated samples presented with the same phenomena.

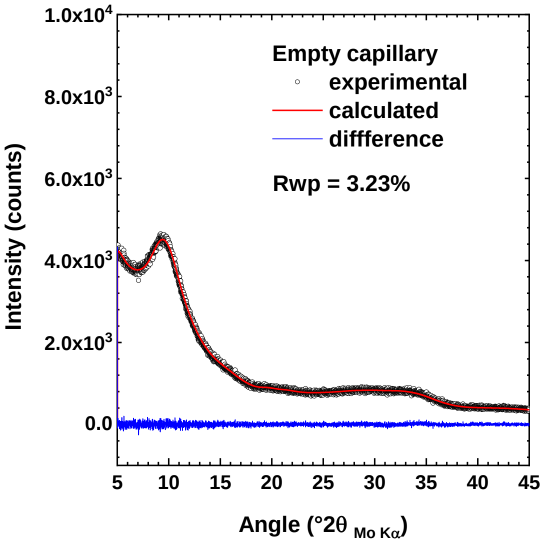

Figure S9. QPA based on full-pattern fitting of HXRD data for an empty capillary.

(a)

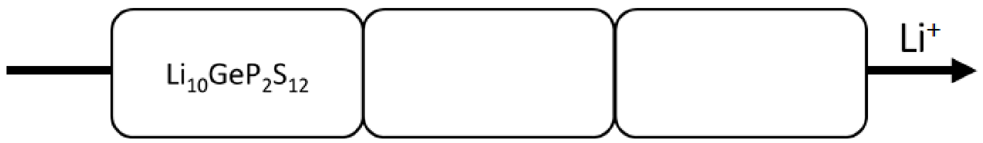

(b)

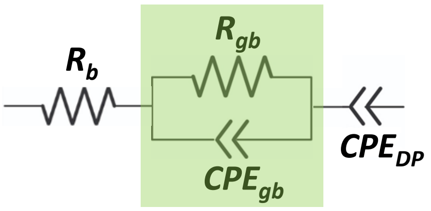

(c)

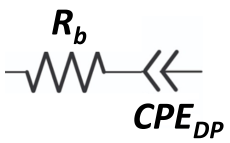

Figure S10. Model of Li-ion transport trough the material that has underwent HBM for 520 min with additional heat treatment at 575 °C (**Fig. S11**) and 600 °C (**Fig. S12**) (a). Equivalent circuit used for fitting the impedance spectroscopy (b-c). For the material heat treated to 575 °C (b) was used for -20 °C to 0 °C, and (c) was used for 10 °C to 60 °C. For the material heat treated to 600 °C (b) was used for -20 °C to 0 °C, and (c) was used for 10 °C to 60 °C. Shaded portions of equivalent circuits are represented in the impendence spectroscopy (**Fig. S11** and **S12**) by semi circles of the same color.

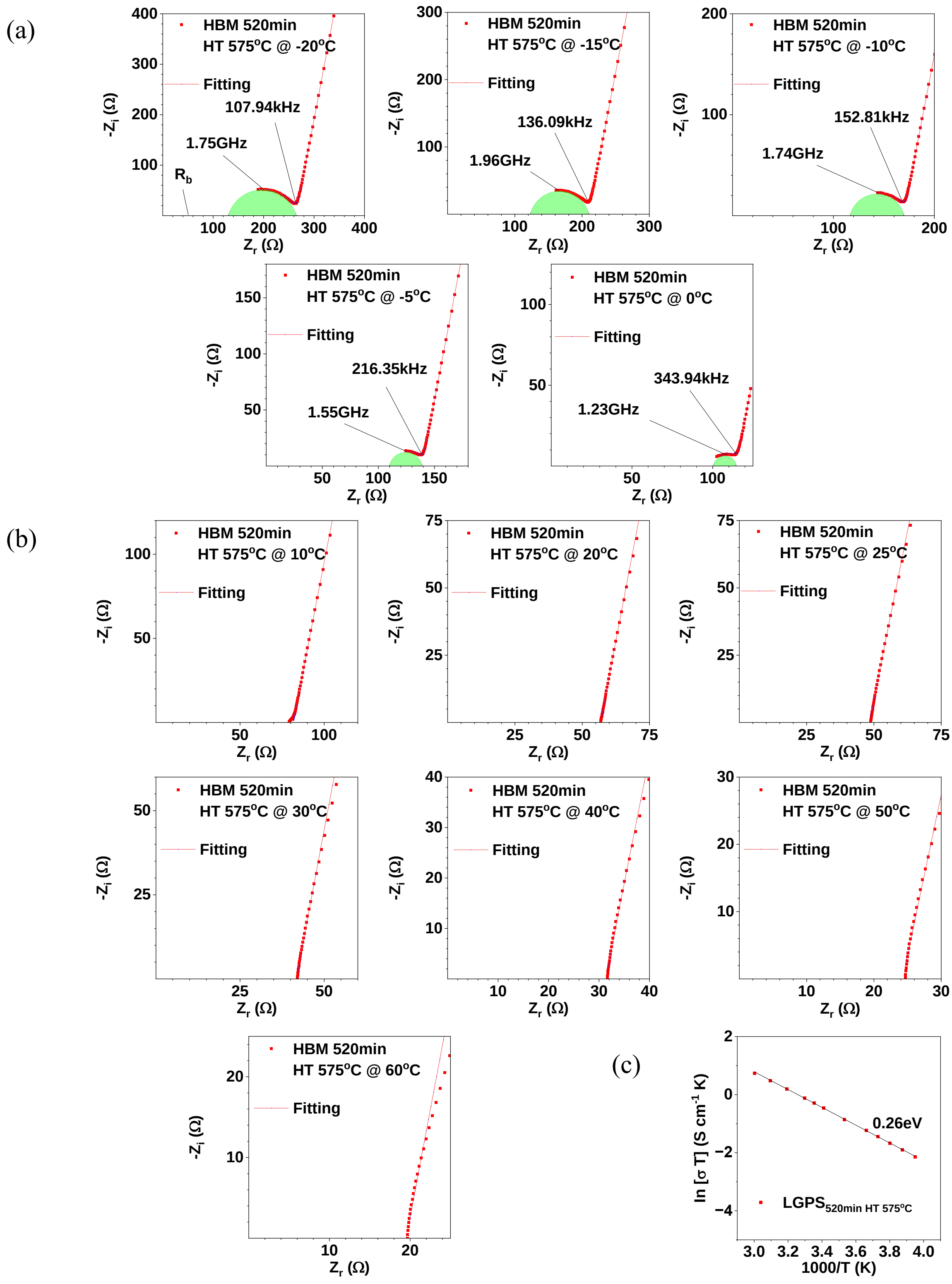

Figure S11. Nyquist plots of sample that has undergone HBM for 520 min with additional heat treatment at 575 °C from -20 °C to 0 °C (a), and 10 °C to 60 °C (b). Equivalent circuit used are found in Figure S10. Arrhenius plot measured from -20 °C to 60 °C along with calculated activation energy (c).

Table S7. Fitting values used for sample that has undergone HBM for 520 min and then a further heat treatment at 575 °C (**Fig. S11**). Where Q_{gb} and a_{gb} describe CPE_{gb}, S_1, and Q_{DP} and a_{DP} describe CPE_{DP}. R_{total} is the sum of all resistors in the circuit.

(°C)	R_b (Ohm)	Q_{gb} (F.s^(a - 1))	a_{gb}	R_{gb} (Ohm)	Q_{DP} (F.s^(a - 1))	a_{DP}	R_{total} (Ohm)	σ (S cm^{-1})
**-20**	130.1	1.056e-8	0.828	134.7	0.578e-6	0.881	264.8	4.60e-04
**-15**	123.1	1.108e-8	0.850	87.03	0.614e-6	0.880	210.13	5.80e-04
**-10**	116.7	1.414e-8	0.866	53.19	0.642e-6	0.880	169.89	7.17e-04
**-5**	110.0	1.877e-8	0.894	28.89	0.672e-6	0.880	138.89	8.77e-04
**0**	100.6	2.577e-8	0.937	13.96	0.719e-6	0.878	114.56	1.06e-03
	**R_b**	**Q_{DP}**	**a_{DP}**				**R_{total}**	**σ**
**10**	81.54	0.768e-6	0.878				81.54	1.49e-03
**20**	56.72	0.816e-6	0.880				56.72	2.15e-03
**25**	48.26	0.874e-6	0.877				48.26	2.53e-03
**30**	41.68	0.860e-6	0.881				41.68	2.92e-03
**40**	31.25	0.995e-6	0.875				31.25	3.90e-03
**50**	24.24	1.176e-6	0.868				24.24	5.03e-03
**60**	19.5	1.094e-6	0.881				19.5	6.25e-03

(a)

(b)

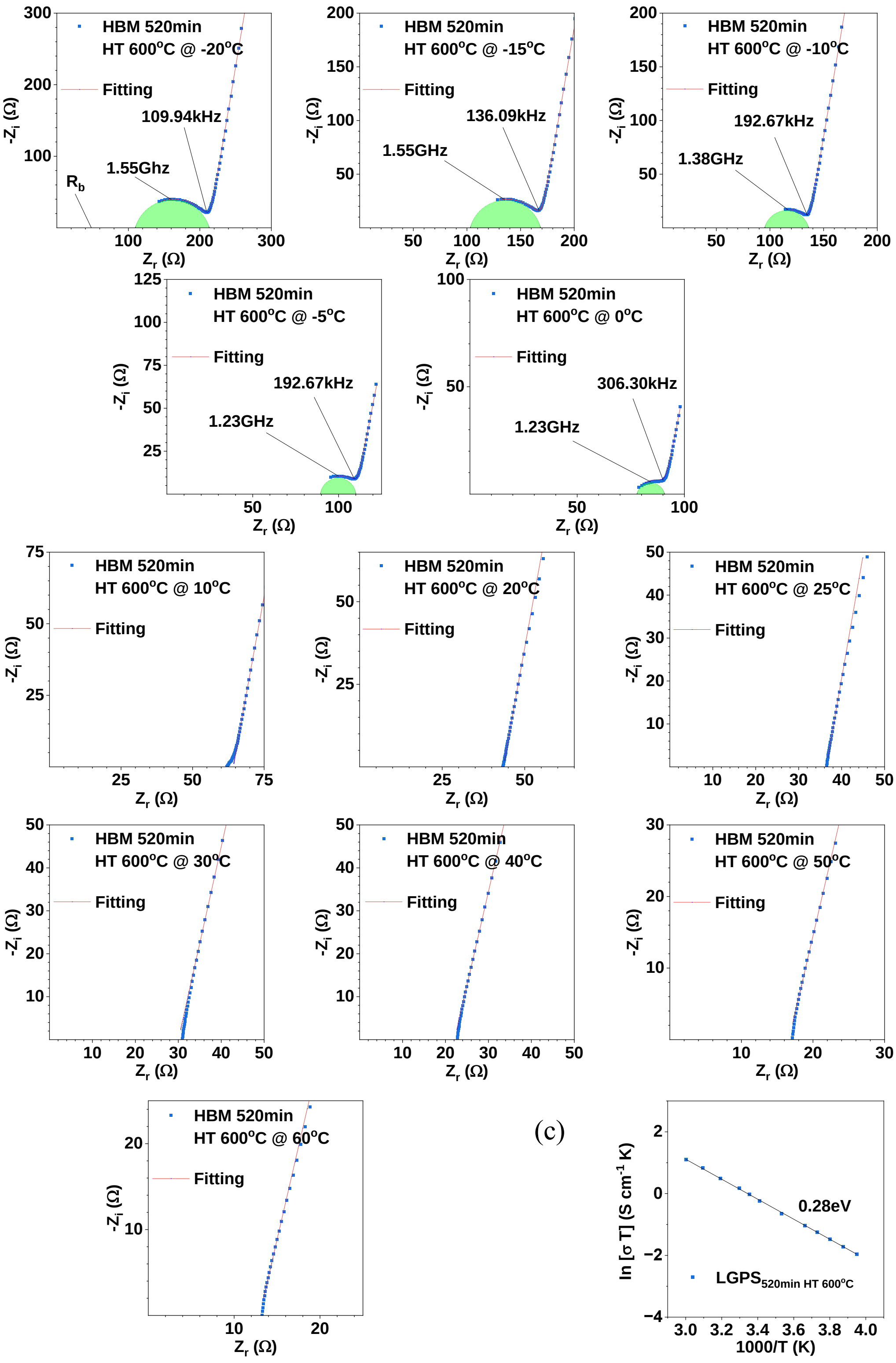

Figure S12. Nyquist plots of sample that has undergone HBM for 520 min with additional heat treatment at 600 °C from -20 °C to 0 °C (a), and 10 °C to 60 °C (b). Equivalent circuit used are found in Figure S10. Arrhenius plot measured from -20 °C to 60 °C along with calculated activation energy (c).

Table S8. Fitting values used for sample that has undergone HBM for 520 min and then a further heat treatment at 600 °C (**Fig. S11**). Where Q_{gb} and a_{gb} describe CPE_{gb} and Q_{DP} and a_{DP} describe CPE_{DP}. R_{total} is the sum of all resistors in the circuit.

(°C)	R_b (Ohm)	Q_{gb} (F.s^(a - 1))	a_{gb}	R_{gb} (Ohm)	Q_{DP} (F.s^(a - 1))	a_{DP}	R_{total} (Ohm)	σ (S cm^{-1})
**-20**	109.8	1.738e-8	0.825	103.4	0.530e-6	0.895	213.2	5.52e-04
**-15**	103.2	1.866e-8	0.847	65.43	0.569e-6	0.893	168.63	6.98e-04
**-10**	95.19	2.817e-8	0.851	40.82	0.587e-6	0.894	136.01	8.65e-04
**-5**	89.5	1.626e-8	0.941	20.55	0.723e-6	0.879	110.05	1.07e-03
**0**	77.9	8.522e-9	0.869	12.81	0.739e-6	0.882	90.71	1.30e-03
	**R_b**	**Q_{DP}**	**a_{DP}**				**R_{total}**	**σ**
**10**	64.14	0.758e-6	0.885				64.14	1.84e-03
**20**	43.49	0.827e-6	0.886				43.49	2.71e-03
**25**	35.98	1.042e-6	0.870				35.98	3.27e-03
**30**	30.02	1.215e-6	0.860				30.02	3.92e-03
**40**	22.09	1.427e-6	0.856				22.09	5.19e-03
**50**	16.63	1.635e-6	0.854				16.63	7.08E-03
**60**	12.96	1.836e-6	0.855				12.96	9.08E-03

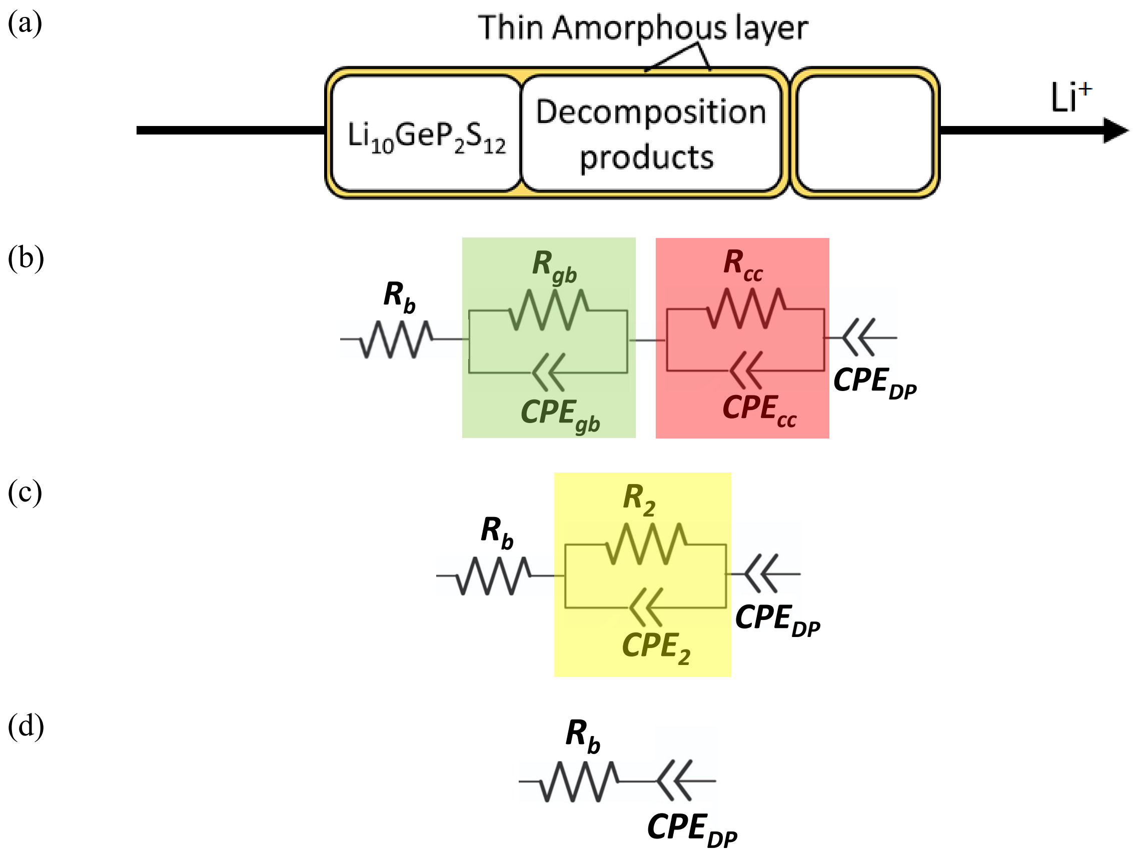

Figure S13. Model of Li-ion transport trough the material that has underwent HBM for 520 min with additional heat treatment at 625 °C (**Fig. S14**) (a). Equivalent circuit used for fitting the impedance spectroscopy (b-d). (b) was used for -20 °C, (c) -15 °C to 30 °C and (d) 40 °C to 60 °C. Shaded portions of equivalent circuits are represented in the impendence spectroscopy (**Fig. S4** and **S5**) by semi circles of the same color.

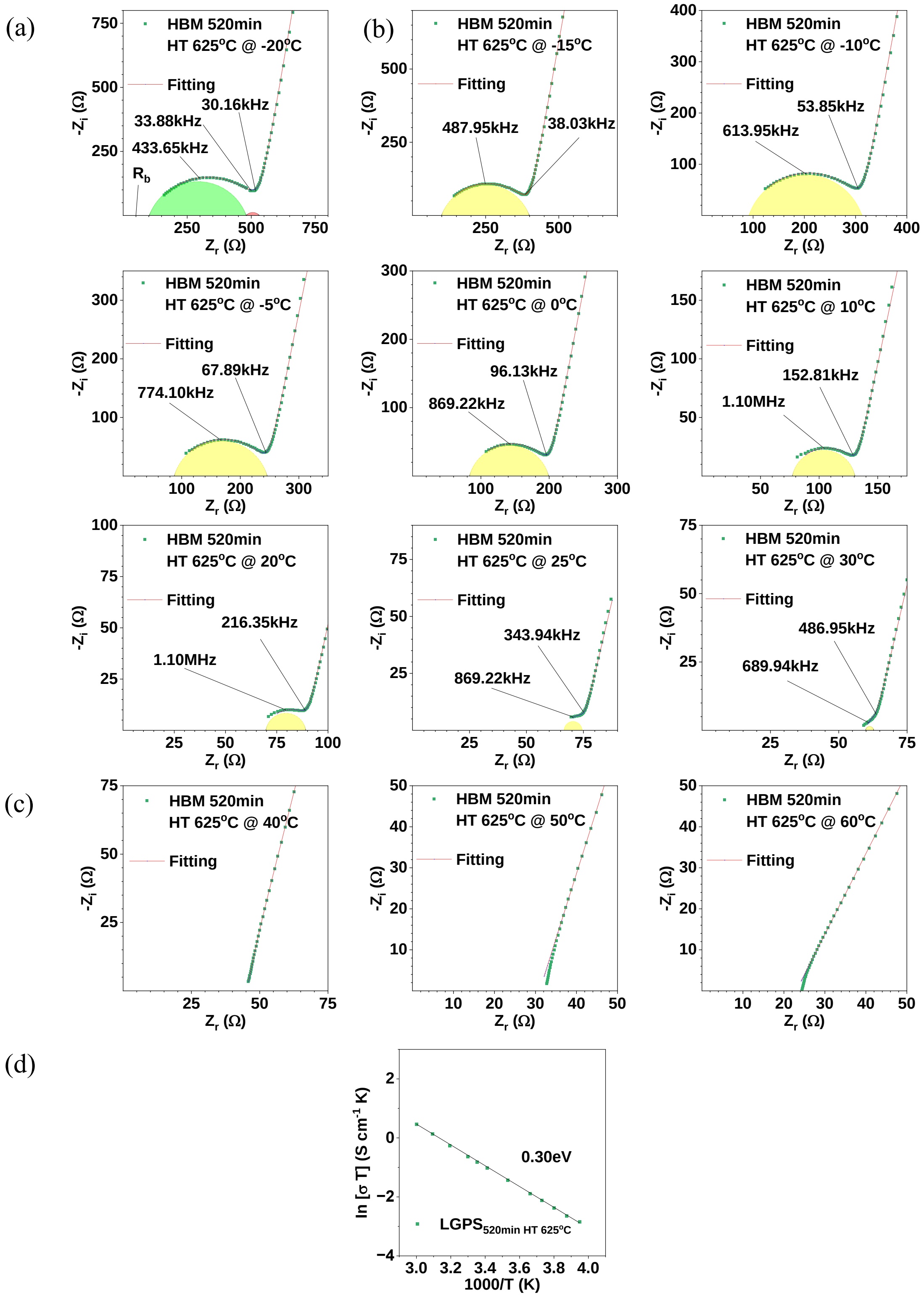

Figure S14 Nyquist plots of sample that has undergone HBM for 520 min with additional heat treatment at 625 °C at -20 °C (a), -15 °C to 30 °C (b), and 40 °C to 60 °C (c). Equivalent circuit used are found in Figure S10. Arrhenius plot measured from -20 °C to 60 °C along with calculated activation energy (d).

Table S9. Fitting values used for sample that has undergone HBM for 520 min and then a further heat treatment at 625 °C (**Fig. S14**). Where Q_{gb} and a_{gb} describe CPE_{gb}, Q_{cc} and a_{cc} describe CPE_{cc}, and Q_{DP} and a_{DP} describe CPE_{DP}. R_{total} is the sum of all resistors in the circuit.

(°C)	R_b (Ohm)	Q_{gb} (F.s^(a - 1))	a_{gb}	R_{gb} (Ohm)	Q_{cc} (F.s^(a - 1))	a_{cc}	R_{cc} (Ohm)	Q_{DP} (F.s^(a - 1))	a_{DP}	R_{total} (Ohm)	σ (S cm^{-1})
**-20**	102	3.078e-8	0.778	378.5	3.482e-6	0.555	51.01	0.389e-6	0.895	480.5	2.29e-04
	**R_b**	**Q_2**	**a_2**	**R_2**	**Q_{DP}**	**a_{DP}**				**R_{total}**	**σ**
**-15**	99.15	3.041e-8	0.777	299.9	0.425e-6	0.890				399.05	2.76e-04
**-10**	91.92	2.578e-8	0.796	220.4	0.452e-6	0.888				312.32	3.53e-04
**-5**	87.39	1.891e-8	0.825	158.6	0.532e-6	0.876				245.99	4.48e-04
**0**	83.41	1.871e-8	0.840	115.8	0.526e-6	0.881				199.21	5.54e-04
**10**	77.21	1.726e-8	0.881	53.31	0.644e-6	0.869				130.52	8.45e-04
**20**	69.7	3.200e-8	0.905	19.44	0.730e-6	0.865				89.14	1.24e-03
**25**	66.58	2.553e-8	1	7.81	0.870e-6	0.854				74.39	1.48e-03
**30**	59.85	1.007e-7	1	2.89	0.901e-6	0.855				62.74	1.76e-03
	**R_b**	**Q_{DP}**	**a_{DP}**							**R_{total}**	**σ**
**40**	44.8	1.061e-6	0.850							44.8	2.46e-03
**50**	30.98	2.155e-6	0.805							30.98	3.56e-03
**60**	22.98	12.56e-6	0.700							22.98	4.80e-03
